# Carbon dot-based fluorescent sensors for pharmaceutical detection: Current innovations, challenges, and future prospects

**DOI:** 10.1016/j.heliyon.2024.e41020

**Published:** 2024-12-06

**Authors:** Sandesh R. Lodha, Jesika G. Merchant, Arya J. Pillai, Anil H. Gore, Pravin O. Patil, Sopan N. Nangare, Gajanan G. Kalyankar, Shailesh A. Shah, Dinesh R. Shah, Shashikant P. Patole

**Affiliations:** a Maliba Pharmacy College, Uka Tarsadia University, Maliba Campus, Gopal Vidyanagar, Bardoli, 394350, Gujarat, India; b Tarsadia Institute of Chemical Science, Uka Tarsadia University, Bardoli, 394350, Gujarat, India; c H.R Patel Institute of Pharmaceutical Education and Research, Shirpur, 425405, Maharashtra, India; d Department of Physics, Khalifa University of Science and Technology, Abu Dhabi, 127788, United Arab Emirates

**Keywords:** Carbon dots, Fluorescent probe, Forster resonance energy transfer mechanism, Quenching, Pharmaceutical detection

## Abstract

Environmental contamination by pharmaceuticals has become a matter of concern as they are released in sewage systems at trace levels, thus impacting biological systems. Increasing concerns about the low-level occurrence of pharmaceuticals in the environment demands sensitive and selective monitoring. Owing to their high sensitivity and specificity carbon dots (CDs) have emerged as suitable fluorescent sensors. This review discusses the current scenario of the status of pharmaceuticals in the environment, limitations associated with traditional techniques employed for their detection, and benefits offered by CDs like easy surface modification and tunable optical properties for sensing applications. Several representative means by which CDs interact with other molecules such as inner filter effect (IFE), dynamic quenching (DQ), static quenching (SQ), Förster resonance energy transfer (FRET), among others, are also discussed along with co-referencing fluorophores to design sensors. Based on developments described herein, CDs-based sensors can be expected to sense pharmaceuticals ranging from nanogram to picogram, target real-time industrial and spiked sample analysis, etc., which provides direction for future research.

## Highlights

• Carbon dots (CDs) are unique fluorescent labels with photoluminescence (PL) properties that make them perfect for sensing.
• Comprehensive comparison of traditional and novel CD-based analytical methods shows CDs' pharmaceutical detection benefits.
• Dopants and surface modifiers affect CD PL characteristics, improving selectivity and sensitivity, according to the study.
• Carbon dots (CDs) detect pharmaceuticals effortlessly, selectively, sensitively, and economically.

## Graphical abstract

**Figure:**
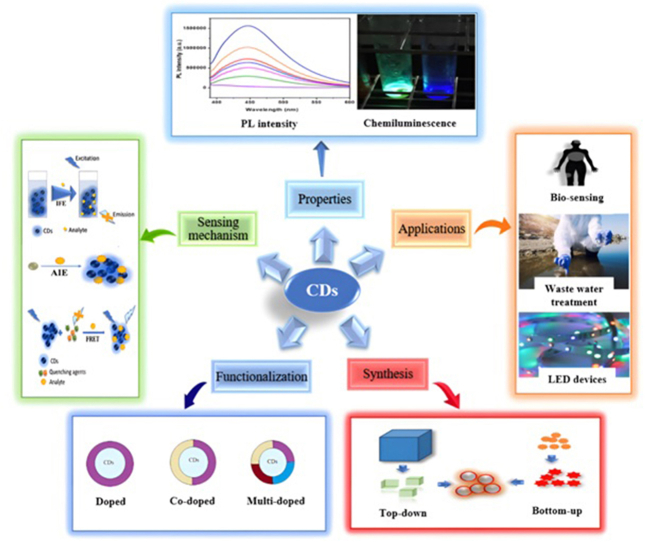

## 1 Introduction

Pharmaceuticals are essential in healthcare for treating various conditions, but they must be administered carefully to avoid adverse effects. Concerns about environmental contamination have risen as pharmaceuticals and their metabolites, classified as “emerging contaminants” for over 15 years, are frequently detected in wastewater, eventually entering natural water sources through industrial discharge and human excretion [1,2]. Developed nations often face issues with lifestyle drug misuse, while developing countries struggle with counterfeit life-saving drugs [3]. Pharmaceuticals in wastewater, with concentrations from micrograms to nanograms per liter, pose risks as current treatment systems fail to remove them entirely, allowing these compounds and their by-products to reach drinking water sources [4]. Moreover, organic solvents in pharmaceutical analysis contribute significantly to environmental and health issues, especially low-boiling-point solvents like n-hexane, toluene, methylene chloride, benzene, chloroform, methanol, ethanol, and acetonitrile, which have known toxic effects [5]. For instance, benzene can cause anemia, hexane is a neurotoxin, and prolonged exposure to chloroform can damage the liver and kidneys. Recognizing the severe impact of these solvents, some are classified as “red solvents” by Pfizer due to their high toxicity [6].

Fluorimetric techniques offer an efficient solution to reduce medication toxicity and solvent use. They require minimal sample preparation and solvent consumption. Simple instruments can detect fluorescent compounds at concentrations up to a thousand times lower than absorption spectrophotometry. By dissolving samples in a suitable solvent, chemical changes can make non-fluorescent compounds detectable, and applicable to both organic and inorganic substances.

Medicines are essential for treating and preventing diseases, and for maintaining public trust in healthcare systems. However, all medications carry a risk of side effects, making it crucial to monitor both intended and unintended outcomes to balance risk and efficacy. Detecting impurities in both active ingredients and formulations is therefore vital [7]. Early identification of unexpected substances, especially in new drugs, is important to reduce health risks. Pharmaceuticals, even at low doses, can be highly active, with contraceptives at nanograms/Liter (ng/L) levels potentially disrupting endocrine systems in municipal water. Excretion and improper disposal release pharmaceuticals into the environment, highlighting the need for effective detection to minimize their environmental and animal toxicity [[7], [8], [9]].

**Fig. 1:**
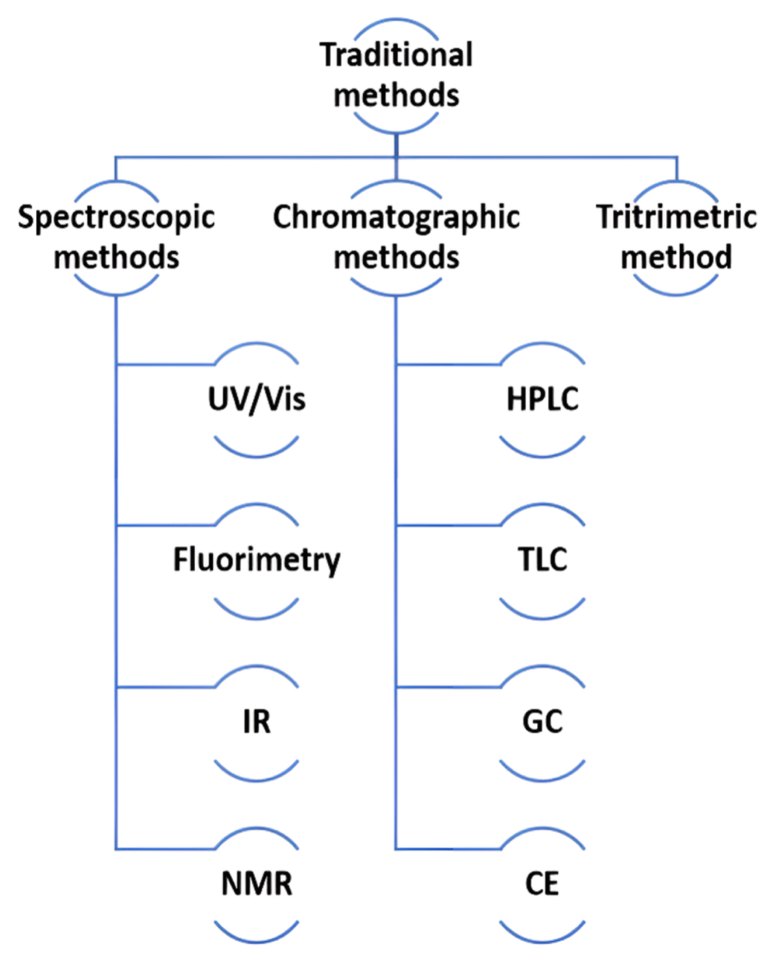
Traditional analytical techniques.

As pharmaceutical research advances, the development of novel and highly selective analytical techniques is crucial for faster, more efficient methods that offer cost savings and reduce solvent consumption. This work evaluates recent quantitative analytical methods and their applications in pharmaceutical analysis, which are essential for quality control and require quick, reliable, and clear results. Key techniques used for the quantitative analysis of pharmaceutical compounds include capillary electrophoresis (CE), high-performance liquid chromatography (HPLC), Ultraviolet–Visible (UV/Vis) spectrophotometry, fluorimetry, titrimetry, voltammetry (in electroanalytical methods), thin-layer chromatography (TLC), gas chromatography (GC), and vibrational spectroscopies (Fig. 1).

UV/Vis spectrophotometry is a widely used method in pharmaceutical analysis, measuring how much ultraviolet (UV) (190–380 nm) or visible (380–800 nm) radiation a chemical absorbs. It works by assessing the relationship between two beams of UV light, where absorption occurs when the energy matches the electronic transitions in the molecule. Combining UV and Vis spectrophotometry offers convenience due to its rapid analysis and ease of use [10,11]. Fluorimeters and spectrofluorometers, which measure fluorescence (FL), offer higher sensitivity compared to other absorption methods. The limitations of FL spectroscopy arise from the sensitivity of detectors to different wavelengths and variations in energy intensity. Infrared (IR) spectroscopy, covering near IR (0.8–2 μm), mid IR (2–15 μm), and far IR (15–1000 μm) ranges, provides detailed structural information of organic and inorganic compounds, with the fundamental 2–15 μm range being most informative for chemical structure analysis [12]. Nuclear Magnetic Resonance (NMR) spectroscopy examines molecular structure at the atomic level, particularly using the ^1^H and ^13^C isotopes, providing detailed structural data through molecular vibrations [13]. High-Performance Liquid Chromatography (HPLC) is an advanced technique that improves upon traditional column chromatography by enhancing separation speed, resolution, accuracy, and sensitivity. HPLC offers benefits like small sample size, customizable tests, and precise data generation, making it a valuable tool in pharmaceutical analysis. Spectrofluorimetry, the most sensitive method, can detect substances at the femtogram level, unlike other methods that require microgram-level sample preparation [14].

This review differs from existing ones by providing a detailed exploration of the synthesis, surface functionalization, and photoluminescent properties of carbon dots (CDs), with a particular emphasis on their emerging applications in photocatalysis, energy, and sensing. Unlike other reviews, we focus on the potential of CDs as safer alternatives to conventional fluorescent compounds, particularly in in-vivo analysis. The specific aims of this review are to offer a comprehensive overview of recent advancements in CDs, highlighting their diverse applications and exploring their potential as substitutes for toxic compounds, as well as their role in electron storage and transport when exposed to light. Furthermore, this review addresses key gaps in the current literature, including the need for alternative materials that can efficiently remove unfiltered contaminants in wastewater treatment and the underutilized potential of CDs in nanomaterial-based solutions. By examining these aspects, we aim to uncover new avenues for the application of CDs in both biotechnological and environmental fields.

Based on the recent advancements in the field of CDs, this review hypothesizes that CDs, due to their unique photoluminescent properties, surface functionalization, and environmental compatibility, can serve as safer and more efficient alternatives to traditional fluorescent compounds in various applications, including sensing, photocatalysis, and environmental monitoring. Furthermore, it is hypothesized that CDs have the potential to overcome the limitations of conventional wastewater treatment systems by offering innovative solutions for contaminant removal, thus providing significant benefits in both biotechnological and environmental fields.

## 2 Nanomaterials in sensing of pharmaceuticals

Nanomaterials are materials that have at least one dimension in the nanometer (nm) range and range in size from approximately 1 to 100 nm [15]. According to the literature, most drug identification methods are based on chromatographic techniques. These techniques are well-established and accepted by regulatory authorities. However, it has several drawbacks related to relatively high cost, analysis time, and pretreatment steps [16]. The incorporation of nanomaterials has enabled the development of novel and effective sensor platforms [17]. The use of nanomaterials for biosensor development has attracted a lot of interest, and carbon nanomaterials (CNMs) are at the forefront. CNMs are composed of sp^2^-bonded graphitic carbon and are mainly classified into fullerenes (0-dimensional), carbon nanotubes (CNTs) (1-dimensional), and graphene (2-dimensional) [18]. CNMs offer superior electrical conductivity, chemical stability, biocompatibility, and strong mechanical strength because of special characteristics including surface-to-volume ratio [19].

As anticipated, these characteristics can affect the stability and selectivity of nanomaterials, as can the capacity to form hydrogen bonds, stacking, dispersion forces, dative bonds, and hydrophobic interactions [20]. A quick search of the Web of Science database over the past five years (2013–2018) for the terms “fullerenes,” “CNTs,” and “graphene” yields over 12,000, 32,000, and 98,000 papers, respectively [21]. The facile functionalization and modification of such nanomaterial-based biosensors facilitated improved efficiency in the detection of antibiotic residues and narrow therapeutic index (NTI) drugs [22]. Additionally, nanoparticles (NPs) can be used for targeted drug delivery [23] and high drug-loading capacities [24]. They also show great potential in cancer therapy by improving drug performance, reducing systemic side effects, and increasing therapeutic efficacy [25]. However, there are complexities, difficult and lengthy synthetic processes, and the toxicity of heavy metal quantum dots (QDs) may hinder their application in biosensing [26]. Therefore, nanomaterials can be used to determine drugs [27]. However, some of them are toxic to humans. A common mechanism by which metal oxide NPs cause toxicity is a combination of the NPs properties and their propensity to generate reactive oxygen species [ROS] and cause toxicity in cells, genes, and neurons.

### 2.1 Carbon dots (CDs)

Quasi-spherical particles having a diameter of less than 10 nm, known as fluorescent CDs were first identified in 2004 [28]. CDs are a new class of nanostructures made of carbon (C) that have intriguing characteristics and small sizes. Surface-functionalized carbonaceous NPs known as CDs have extraordinary properties, including adjustable FL [29]. CDs were reported to have good biocompatibility, less toxicity [30], high photoluminescence (PL) intensity [31], high chemical stability [32], and possess excellent biological, physical, and chemical properties, thus having great potential in various applications [33,34]. Only a few of the benefits associated with their luminescence are their outstanding water solubility, biocompatibility, non-toxicity, high sensitivity to the environment, and apparent electron-donating and receiving capacities [35]. CDs exhibit appealing optical characteristics such as size-dependent PL [36], photo-induced electron transfer (PET), up-conversion luminescence, chemiluminescence, and electrochemiluminescence (ECL) [37]. These dots possess an inner sp^2^ and outer sp^3^ hybridized structure that frequently contains oxygen-containing functional groups. The surface of CDs imparts several traits to them, including effortless electron transfer, which can confer anti- or pro-oxidant behavior [38].

In addition, CDs can be easily functionalized with hydroxyl, carboxyl, carbonyl, amino, and epoxy groups on their surfaces. This additional advantage enables them to bind readily with both inorganic and organic moieties. The functional groups present on the surfaces of CDs allow them to adopt either hydrophilic or hydrophobic properties, providing the necessary thermodynamic stability in different solvents, particularly in water [39]. Moreover, CDs have exceptional sensing properties like multiplex, selective, and specific detectability. Abundant functional groups (such as amine, carboxyl, hydroxyl, etc.) or polymer chains on the surface of CDs make them highly soluble in aqueous solutions and easily functionalized with other nanomaterials [40]. Because of their extremely sensitive responses to target molecules and tunable surface functional groups, these characteristics make CDs particularly appealing in sensing applications. Another fascinating property of CDs is their tunable emission, characterised by multiple FL colors under various excitation wavelengths [41].

### 2.2 Properties of CDs

**Fig. 2:**
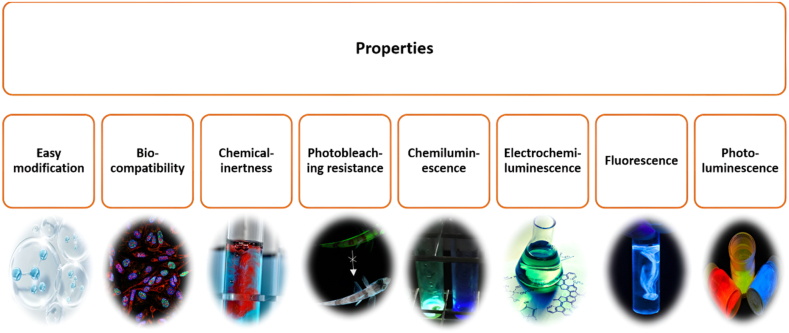
Properties of CDs.

Without a crystal structure, CDs are always spherical and split into carbon NPs. All CDs have linked or altered chemical groups, like chains made of oxygen, amino acids, polymer etc., on their surfaces. X-ray diffraction (XRD), Raman spectroscopy, and high-resolution transmission electron microscopy (HRTEM) are the direct characterization techniques for the carbon core XRD. NMR, X-ray photoelectron spectroscopy (XPS), Fourier transforms infrared (FTIR), and matrix-assisted laser desorption ionisation time-of-flight (MALDI-TOF) are used to assess the grafting of chemical groups. These luminous CDs are therefore not made of “pure” C compounds. Various properties of CDs are depicted in Fig. 2. The PL behavior of these CDs is mostly determined by the hybridization and coupling between the C core and surrounding chemical groups [42]. In the UV spectral region, CDs typically exhibit considerable absorption with an extension into the visible spectrum. Sometimes, at a wavelength that is significantly longer than the UV absorption peak, a shoulder or a weak peak is also seen. Longer wavelength UV absorption, which typically takes the form of a shoulder or a weaker peak between 300 nm and 400 nm, is attributed to n → π∗ transitions of C=O. Shorter wavelength UV absorption, which is roughly between 200 nm and 350 nm, is attributed to π → π∗ electronic transitions of C=C and C=N [43]. The UV–Vis spectra of Yellow-Green CDs (YG-CDs) give several absorption bands at 270 nm and 382 nm, just like Blue-CDs (B-CDs) do. However, YG-CDs have a higher absorption intensity than B-CDs, which may be due to the latter's higher degree of carbonization in a more acidic hydrothermal environment [44]. The PL emission, comprising excitation-dependent and excitation-independent PL, which is caused by core-related and surface state-related emissions, is one of the CDs most fascinating characteristics [45]. Further remarkable FL characteristics of CDs include tunable PL emission, excitation wavelength-dependent PL emission, strong FL stability, and effective photobleaching resistance. Some CDs can have up-conversion PL (UCPL) emission characteristics, which means that the emission wavelengths of carbon quantum dots (CQDs) are shorter than their excitation wavelengths, in contrast to standard PL emission. The most common explanations for the up-conversion FL phenomenon are often two-photon excitation and anti-Stokes PL emissions [46]. CDs exhibit ECL capabilities and are useful for metal ions detection, optical devices, and biosensors [47]. CDs have a protracted, intense FL emission (up to a year). As CDs can withstand a wide pH range (from 3 to 12), they exhibit excellent photobleaching impedance. Excitation following direct oxidation, amplification, or inhibition of luminescence are all ways that CDs might produce chemiluminescence [40].

### 2.3 Synthesis of CDs

**Fig. 3:**
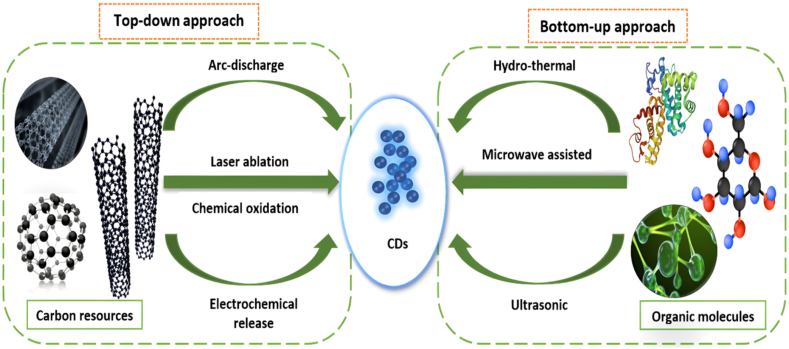
Schematic diagram showing various sources and methods for synthesis of CDs.

There are currently many different synthetic approaches that can be used to make CDs. The majority of studies focus on easy, affordable, size-controllable, or large-scale synthetic ways to produce CDs of superior quality. Chemical and physical processes are used to create synthetic materials, respectively, depending on the characteristics of the transformations of C sources to final products. Moreover, two classes of top-down and bottom-up may characterise existing synthetic techniques when taking into account the link between the sources and products (Fig. 3). The synthesis process may also require additional purification of the end products using techniques like centrifugation, electrophoresis, dialysis, etc. One study by Zhu et al., for instance, used three cycles of concentration/dilution to separate CDs from other reactants [48]. For this class, CDs are produced through oxidation, laser ablation, arc discharge, and electrochemical release on somewhat macroscopic C structures including CNTs, graphite columns, graphene, suspended C powders, etc [49]. In bottom-up processes, CDs are made from a variety of small molecule precursors; most of them enable the manufacture of functionalized CDs in a single step. It consists of pyrolysis, microwave synthesis, ultrasound-assisted synthesis, and hydrothermal synthesis [50]. Several C sources are utilized, including amino acids, citric acid, sugar, and even food waste.

#### 2.3.1 Top-down methods

Chemical oxidation frequently involves oxidising the substrate with an oxidative reagent. Chemical oxidation is also a quick and efficient approach to large-scale production. Coal, wood, and coconut activated carbons-all readily available in the marketplace-were the C sources. Nitric acid made it simple to etch CDs out of the amorphous structure of activated carbons. Chemicals with amine ends were then used to carry out the passivation process. The three C sources' end products had a constrained size distribution and spectacular luminosity [[51], [52], [53]]. Because a huge volume of fluorescent CDs can be created in a short period, the laser ablation including solid targets in liquid (LASL) approach for producing fluorescent CDs can be very rapid and efficient. For the synthesis of high-quality or smaller-sized nanostructures, other synthesis procedures, that include the ablation of suspended micro particles or powders, need a longer ablation period. In this technology, greater control of the size distribution can be achieved by carefully controlling heat dissipation via wavelength and intensity change. In comparison to microscale-ablated particles, the size of the emitting NPs generated in the study is in the nanoscale range [51]. Top-down electric arc or arc-discharge synthesis was used to create the first luminous CDs. In order to extract fluorescent C from raw small wall nanotubes (SWNTs), soot, and other materials using a preparative electrophoretic method, it was shown that the fluorescent materials PL quantum yield (PLQY) could reach 1.6 percent at 366 nm excitation wavelength. The benefit of this approach is that the CDs may emit multiple fluorescent colors under UV light without undergoing any surface alteration, and it just needs a simple purification procedure. It is also portable and environmentally friendly. The formation of mixes, low yield, difficulties in surface and compositional modification, and difficulty in these alterations are also drawbacks [52]. A C material, such as graphite, CNTs, or C fibre electrodes, is chemically cut using the electrochemical method, which is influenced by an electric field. The method's low cost and simplicity make it useful. Interestingly, by altering process variables like the temperature, electric field, CNT diameter, and concentration of the supporting electrolyte, CDs made electrochemically can have their sizes adjusted [53].

#### 2.3.2 Bottom-up methods

A simple bottom-up hydrothermal approach has been reported to make CDs. The colorless solution first needs to close in a Teflon-coated stainless-steel autoclave and then be kept at a definite temperature for a particular time. The autoclave was left to naturally cool to room temperature following the reaction. The solution is either centrifuged or passed through dialysis tubing of a certain molecular weight to remove the precipitation and to eliminate leftover small molecules, the CDs were dialyzed against ultrapure water at room temperature using a membrane (MCWO 1000 Da) [54]. Depending on the frequency and strength of the applied field, ultrasound is a typical laboratory instrument that can be used to emulsify mixtures, drive chemical processes, and nebulize liquids into fine mists [55]. Microwaves are electromagnetic wave types with a broad wavelength range of 1 mm (mm) to 1 m (m) that are frequently employed in daily life and science. The microwave can also deliver high energy to break the chemical bonds in the substrate, just like a laser can. The synthesis of CDs is thought to be more energy-efficient when done in a microwave, and the reaction time can also be significantly reduced. The substrate is typically pyrolyzed and surface functionalized during microwave-assisted synthesis [56].

### 2.4 CDs as a sensor

**Fig. 4:**
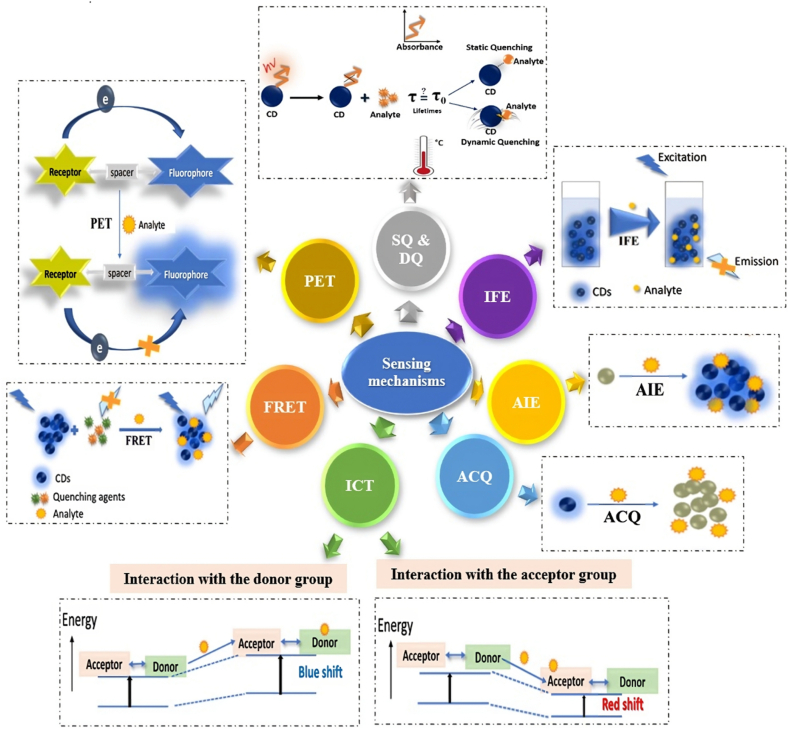
The sensing mechanism of CDs.

The sensing mechanism of the CDs depends upon the chemical bonding of the fluorescent probe. Various mechanisms of CDs have been available (Fig. 4) for sensing the analyte, these include Förster resonance energy transfer (FRET) [57,58], inner filter effect (IFE) [59,60], photoinduced electron transfer (PET) [61,62], intramolecular charge transfer (ICT) [63,64], static quenching (SQ), dynamic quenching (DQ) [65,66], aggregation caused quenching (ACQ), aggregation-induced emission (AIE) [67].

#### 2.4.1 Different mechanisms of CD

The FL behavior of CDs is governed by various mechanisms that influence their emission properties and sensing capabilities. These mechanisms include FRET, IFE, SQ and DQ, PET, ICT, as well as phenomena like ACQ and AIE.

#### 2.4.2 Förster resonance energy transfer (FRET)

In FRET, energy is transferred non-radiatively from an excited donor molecule (D), typically a fluorophore, to a close-by ground-state acceptor molecule (A) via long-range dipole-dipole interactions. For efficient FRET, the acceptor must absorb energy at the donor's emission wavelength (s), though it does not need to emit this energy fluorescently. The efficiency of FRET is significantly influenced by three main factors: the degree of spectral overlap between the donor's emission and the acceptor's absorption, the relative orientation of the transition dipoles, and most importantly, the distance between D and A, usually in the range of 10–100 Å (Å), which is comparable to the size of many biological macromolecules [57,58].

#### 2.4.3 Inner filter effect (IFE)

The IFE involves an absorber in a sensor system that modulates the excitation or emission of light from a fluorescent molecule. In an IFE-based sensing system, both an absorber and a fluorescent molecule must coexist. The absorber's absorption spectrum overlaps with the excitation and/or emission spectra of the fluorophore. This overlap enables the absorber to control and influence the FL emission. The system requires the absorber to exhibit a selective response to the analyte concentration, while the FL intensity of the fluorophore remains unaffected by the analyte, ensuring its role as an indicator in the sensor system [59,60].

#### 2.4.4 Photoinduced electron transfer (PET)

PET is a mechanism in which a fluorophore is linked to a recognition receptor via a spacer. FL quenching in PET occurs due to intramolecular electron transfer between the receptor and the fluorophore. Two types of PET processes are recognized: (1) Acceptor-PET (A-PET): Electron transfer from the receptor to the fluorophore occurs when the receptor's Highest Occupied Molecular Orbital (HOMO) energy level is higher than that of the fluorophore. (2) Donor-PET (D-PET): Electron transfer from the excited fluorophore to the receptor's Lowest Unoccupied Molecular Orbital (LUMO) level occurs during D-PET. The PET process is inhibited when the receptor binds to a target analyte, leading to restored FL emission [61,62].

#### 2.4.5 Intramolecular charge transfer (ICT)

ICT involves electron transfer from a donor (D) to an acceptor (A) within a fluorescent probe upon light stimulation. When the donor interacts with an analyte, its electron-donating ability decreases, increasing the HOMO-LUMO energy gap and causing a blue shift in FL emission. Conversely, the interaction of the acceptor with the analyte decreases the energy gap, resulting in a red shift. Thus, both FL intensity and emission wavelength can be used for analyte detection. The varying dipole moments between the ground and excited states of ICT probes make them sensitive to different solvent environments, making them ideal for detecting changes in solvent conditions [63,64].

#### 2.4.6 Quenching mechanisms

##### **2.4.6.1** Static quenching (SQ)

SQ occurs when a non-fluorescent ground-state complex forms between the quencher and the CDs. This complex reduces the FL intensity of the CDs and alters their absorption spectrum. Temperature increases can destabilize this ground-state complex, reducing the quenching effect.

##### 2.4.6.2 Dynamic quenching (DQ)

DQ involves the interaction between an excited-state fluorophore and a quencher, resulting in energy or charge exchange that returns the fluorophore to its ground state. Unlike SQ, DQ affects only the excited state and does not alter the absorption spectrum of the fluorophore. The FL lifetime of the fluorophore is reduced in the presence of a quencher. Higher temperatures typically enhance the DQ effect, increasing the rate of quenching interactions [65,66].

The reduction in FL intensity due to quenching can be quantified using the Stern-Volmer equation:

F_0_/F = 1 + K[Q] = 1 + k_q_τ_0_[Q]

Where K is the Stern-Volmer quenching constant, [Q] is the quencher concentration, τ_0_ is unquenched lifespan, and k_q_ is the bimolecular quenching constant, and F and F_0_ are FL intensities in the presence and absence of the quencher, respectively.

##### 2.4.6.3 Aggregation-caused quenching (ACQ) and aggregation-induced emission (AIE)

In ACQ high concentrations or solid-state fluorophores often exhibit diminished FL due to self-quenching. This effect arises from interactions such as hydrophobic effects, stacking, and hydrogen bonding, leading to non-radiative decay pathways. In contrast to ACQ, AIE materials show enhanced FL in high concentrations or solid forms. In dilute solutions, non-radiative energy dissipation occurs via intramolecular rotation. However, in the aggregated state, this rotation is restricted, leading to suppressed non-radiative decay, and enhanced FL emission organization groups similar principles together, improving the clarity and logical flow of the discussion on various FL mechanisms associated with CDs [67].

### 2.5 Modification of CDs

**Fig. 5:**
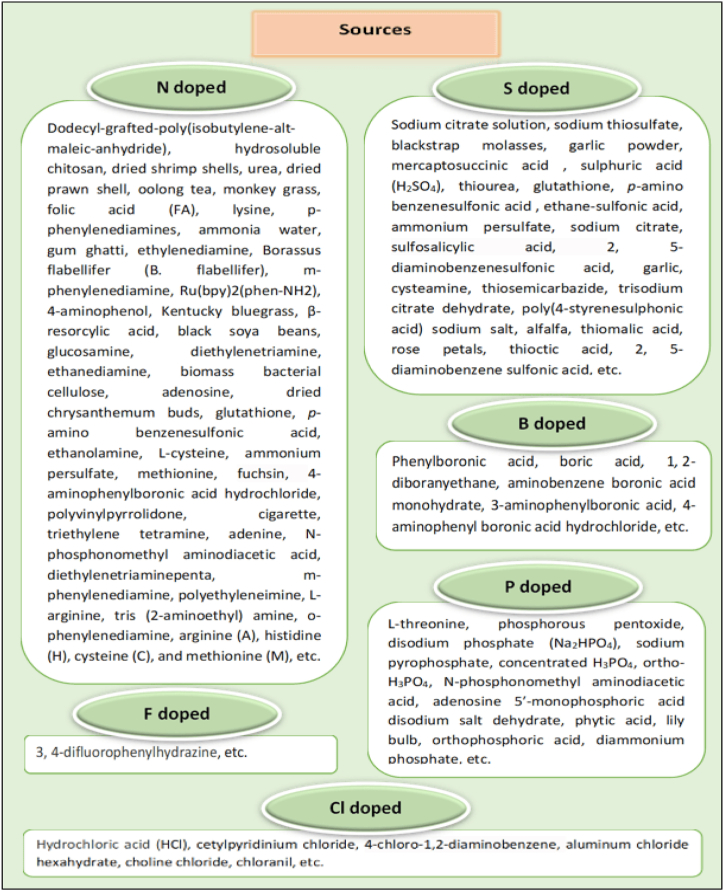
Doped CDs sources.

**Fig. 6:**
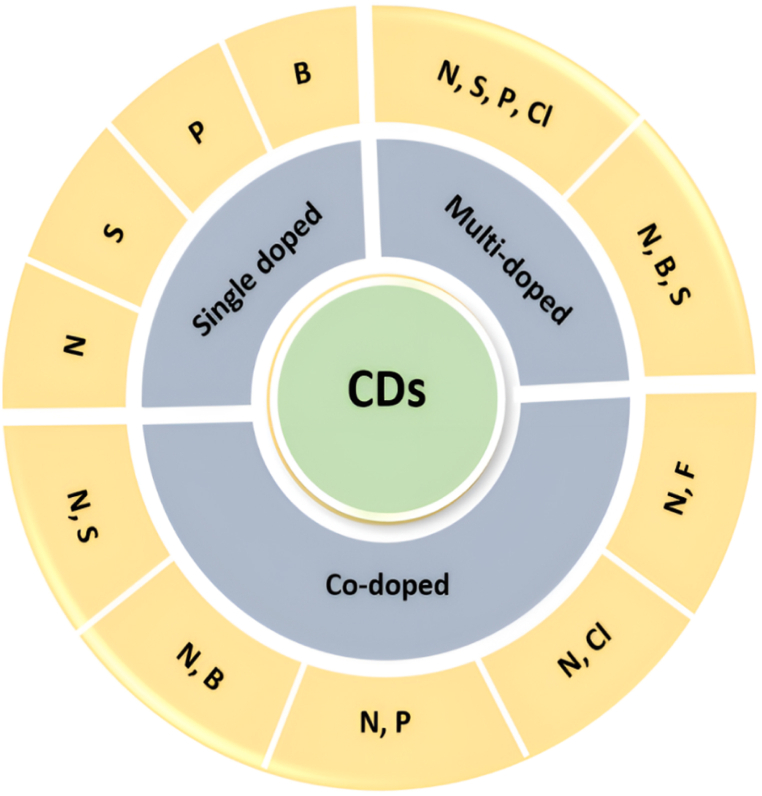
Single, multi, and co-doped CDs.

Doped-CDs are carbogenic NPs with an average size of less than 10 nm that have atomic impurities added like nitrogen (N), sulfur (S), phosphorus (P), boron (B), etc. (Fig. 5) during the production process to enhance their optical, electrical, and chemical capabilities. One can distinguish between single- and multiple-doped CDs (Fig. 6) based on how many atomic impurities have been added to the structure of the CDs. One of the most well-known options is N since it has five valence electrons, a similar atomic size to C, and can form bonds with C atoms. When the N content in the CDs rises, the FL peak has shown that it will move to a longer wavelength under the same stimulation. The observation's justification is based on the N-doped material forming new FL origins. In other research, N is also seen to improve the efficacy of emissions as opposed to displacing them. The mechanism is thought to involve electrons in the conduction band and the occurrence of an upward shift in the Fermi level. Hence, various polymers used as a precursor material for C and N sources are dodecyl-grafted-poly(isobutylene-alt-maleic-anhydride) [68], hydrosoluble chitosan [69], dried shrimp shells [70], citric acid, and urea [71], dried prawn shell [72], oolong tea [73], monkey grass [74], folic acid (FA) and phosphoric acid (H_3_PO_4_) [75], lysine and ortho-phosphoric acid [76], p-phenylenediamines and ammonia water [77], gum ghatti and ethylenediamine [78], Borassus flabellifer (B. flabellifer) and aq. Ammonia [79], m-phenylenediamine [80], Ru(bpy)2(phen-NH_2_) [81], 4-aminophenol [82], ethylenediamine, and Kentucky bluegrass [83], β-resorcylic acid and ethylenediamine [84], black soya beans [85], glucosamine and ethylenediamine [86], citric acid and diethylenetriamine [87], highland barley as C source and ethanediamine as N resource [88], biomass bacterial cellulose [89], tartaric acid and urea [90], adenosine [91], dried chrysanthemum buds, and ethylenediamine [92]. The band-gap energy of photoexcited electrons may be modified by the S atom's density of states or emissive trap states, giving functionalized CDs additional favorable properties [[93], [94], [95], [96], [97], [98], [99], [100], [101], [102]], sodium citrate solution, and sodium thiosulfate [101], blackstrap molasses, H_2_O_2_, and garlic powder [103], mercaptosuccinic acid [104], sulphuric acid (H_2_SO_4_) [105].

Thus various polymers used as precursor material for C and S sources are sodium thiosulfate and sodium citrate [106]. With only three valence electrons, B (one less than C). As a result, adding a B atom to a C cluster causes the generation of p-type carriers inside CDs, altering their electrical structures and optical characteristics [[107], [108], [109], [110], [111]]. Thus various polymers used as precursor material for C and B sources are phenylboronic acid [109], boric acid, urea and citric acid [110], glucose and boric acid [112], L-ascorbic acid and boric acid [113], citric acid, H_2_SO_4_, ammonia water, boric acid, and isopropyl alcohol [114], boric acid and ethylenediamine [115], citric acid monohydrate, thiourea, and boric acid [116]. Moreover, citric acid, rhodamine B, methylene blue,1, 2-diboranyethane, N, N-dimethylformamide (DMF, anhydrous, 99.8 %) [117], etc. The P atom can have a significant impact on the chemical and electrical structures of semiconductors since it is a major electron donor in semiconductors, which are endowed with promising uses in photovoltaic devices and catalysts. The bigger size of the P atom compared to the C atom directly contributes to an increase in disorders inside the P-doped graphite carbon backbone [[118], [119], [120], [121]].

The various polymers used as precursor materials for C and P sources are L-threonine and phosphorous pentoxide [122], sucrose and H_3_PO_4_ [123], dextrose and disodium phosphate (Na_2_HPO_4_) [124], beta-cyclodextrin and sodium pyrophosphate [125], lactose, and concentrated H_3_PO_4_ [126], orthophosphoric acid and glucose [127]. Nitrogen, Sulfur co-doped carbon dots (N, S-CDs), one of the heteroatom-containing CDs, display their remarkable FL behaviors [128]. Example: thiourea and glutathione [129], *p*-aminobenzenesulfonic acid [130], DL-malic acid, ethanolamine and ethane-sulfonic acid [131], citric acid and L-cysteine [132], ammonium persulfate, glucose, and ethylenediamine [133], citric acid and thiourea [134], sodium citrate and thiourea [135], methionine and citric acid [136], basic fuchsin and sulfosalicylic acid [137], 4-aminophenylboronic acid hydrochloride and 2, 5-diaminobenzenesulfonic acid [138], ethylenediamine, L-cysteine and malic acid [139], citric acid and dithiooxamide [140], garlic [141], citric acid monohydrate and cysteamine [142], urea and H_2_SO_4_ [143], citric acid and thiosemicarbazide [144], trisodium citrate dehydrate and L-cysteine [145], H_2_SO_4_ [146], methionine and acrylic acid [147], polyvinylpyrrolidone and poly(4-styrenesulphonic acid) sodium salt [148], citric acid and arginine (A), histidine (H), lysine (L), cysteine (C), and methionine (M) [149]. In addition, other combination includes heating egg white, egg yolk, pigeon feathers or pigeon manure [150], garlic and alfalfa [151], thiomalic acid and diethylenetriamine [152], rose petals, ethylenediamine, L-cysteine [153], para-benzoquinone and L-cysteine [154], cigarette and H_2_SO_4_ [155], thioctic acid and triethylene tetramine [156].

Heteroatoms like N and B have been created to enhance the optical performance of CDs. The luminous characteristics of these CDs are fascinating, including temperature- and pH-dependent FL responses and excellent quantum yield [157]*. Solanum betaceum* (*S. betaceum*) fruit extract [158], trisodium citrate, urea, and boric acid [159], adenine and aminobenzene boronic acid monohydrate [160], citric acid, boric acid and ethylenediamine [161], 2,5-diaminobenzenesulfonic acid and 4-aminophenyl boronic acid [138], petroleum coke [162], 3-aminophenylboronic acid [163], citric acid, boric acid, and tris base [164], citric acid, urea, and boric acid, etc. are the various precursors used for the synthesis of N, B -doped CDs [165]. On the periodic table, N and P sit next to C and serve as important tracking elements in the field of biomedical imaging. These two components change the optical and electronic characteristics of CDs and advance our fundamental knowledge of their PLQY. Moreover, this may result in multifunctional applications in photothermal therapy (PTT) and photoimaging. Some precursors used for the synthesis of N, P-doped CDs are ethylenediamine and N-phosphonomethylaminodiacetic acid [166], 1,4-naphthalenedicarboxylic acid and urea [167], glucose, ammonia, and H_3_PO_4_ [168], adenosine 5′-mono phosphoric acid disodium salt dehydrate [169], diethylenetriaminepenta and m-phenylenediamine [170], glucose, polyethyleneimine and H_3_PO_4_ [171], phytic acid and L-arginine [172], 1,2-ethylenediamine, phthalic acid and H_3_PO_4_ [173], lily bulb [174], citric acid, tris (2-aminoethyl) amine and orthophosphoric acid [175], sodium citrate and diammonium phosphate [176], maize and urea [177], alendronate sodium [178], polyethylene glycol (PEG) (C source), and H_3_PO_4_ [179]. Various sources used for the synthesis of N, Cl-doped CDs are o-phenylenediamine, hydrochloric acid (HCl) and selenourea [180], cetylpyridinium chloride [181], 4-chloro-1,2-diaminobenzene and dopamine [182], p-phenylenediamine, melem hydrazine, and aluminum chloride hexahydrate [183], glycerine, choline chloride and urea [184], chloranil and triethylenetetramine [185], glucose, concentrated HCl and 1,2-ethylenediamine [186]. B, N, and S-doped were synthesized by 2, 5- diaminobenzene sulfonic acid, and 4- aminophenyl boronic acid hydrochloride [187]. The N, S, P, and Cl-doped were synthesized by glucose and ethylenediamine, and then H_2_SO_4,_ concentrated H_3_PO_4_, and concentrated HCl were added orderly into the beaker to get the CDs [188].

## 3 Applications of fluorescent CDs based sensors for pharmaceuticals

As CDs are reported best for sensing pharmaceuticals. Fig. 13 illustrates a variety of CDs applications along with drug identification in pharmaceuticals. So far, some of the drugs listed in Table 1 are detected by carbon nanodots (CNDs) through a different mechanism of CDs. The study investigated the impact of deoxygenation and radical scavengers on the FL intensity of the C-dots-Ni(IV) system. It was proposed that when Ni(IV) reacts with dissolved oxygen in an alkaline solution, it produces the superoxide radical (•O_2_^−^). Both Ni(IV) and •O_2_^−^ can interact with C-dots, generating C-dot•^+^ and C-dot•^−^, which then undergo electron transfer annihilation, forming an excited-state C-dot∗ as the final emitter. The target analyte was found to inhibit chemiluminescence by competitively reacting with Ni(IV). This led to the development of a flow-injection chemiluminescence method, which proved to be a fast, simple, sensitive, and robust technique for detecting trace levels of reducing compounds [189]. In a separate experiment, CDs were synthesized using sodium citrate and ammonium bicarbonate through a hydrothermal process. The FL intensity of the CDs was found to be pH-dependent, increasing with pH up to 6.0, before stabilizing between pH 7.3 and 7.7. The study also tested the effects of various interfering substances, revealing that D-penicillamine (D-PA) caused significant quenching of the FL, while increasing concentrations of Hg^2+^ also reduced the FL intensity, with near-total quenching occurring at 4 × 10⁻⁵ mol L⁻^1^ of Hg^2+^ [190].

**Table 1:** Pharmaceutical applications of fluorescent CDs-based sensors.

Sr. No.	Drug name	Wavelength excitation(λ_ex_)	Wavelength emission(λ_em_)	Linearity range	Mechanism	Ref
1.	Paracetamol	360 nm	445 nm	4.48x10^−7^-8.96 x10^−5^ μM	Ni(IV) reacted with CDs produced could form an excited state C-dot∗ which interacts with the target analyte due to its competitive reaction with Ni(IV).	[189]
2.	D-penicillamine	358 nm	442 nm	2–24 μmol/L	Fluorescent switch sensor	[190]
3.	Kaempferol	370 nm	490 nm	3.5–4.9 μM	Non-radiative energy transfer (FRET)	[191]
4.	Amantadine	335 nm	446 nm	–	FRET	[192]
5.	Chloramphenicol	400 nm	525 nm	–	FRET	[192]
6.	Hyperin	300 nm	540 nm	0.22–55 μM	Non-radiative energy transfer	[193]
7.	Sumatriptan	380 nm	446 nm	1–20 μM	The fluorescent quenching observed is caused by the formation of hydrogen bonds between the nitro groups of DNB-CDs and the aliphatic -NH group on the phenyl aromatic ring of SUM.	[194]
8.	6-Mercaptopurine	370 nm	445 nm and 565 nm	1.0–100.0 μmol L^−1^	FRET	[195]
9.	Gentamicin	340 nm	385 nm	0 to 2.9 × 10^−4^ mol/L	FRET	[196]
10.	Gentamicin and kanamycin	390 nm	482 nm	200–2000 nM	FRET	[197]
11.	α-glucosidase	410 nm	510 nm	100 μL	IFE	[198]
12.	Iron	370 nm	432 nm	0–1.5 mM	IFE	[199]
13.	Hematin	314 nm	362 nm	0–75 μM	IFE	[200]
14.	Cefixime	350 nm	455 nm	0.2 × 10^−6^ M to 8 × 10^−6^ M	IFE	[201]
15.	Tetracycline	345 nm	435 nm	0.5–25 μM	IFE	[202]
16.	Chlortetracycline	370 nm	447 nm	0.4–20 μg/mL	IFE	[203]
17.	Tetracycline	350 nm	420 nm	0–50 μM	IFE	[204]
18.	Tetracycline	370 nm	440 nm	0.5–60 μM	Internal filtering effect	[205]
19.	Tetracycline	360 nm	440 nm	0.1–4.0 μg mL^−1^	IFE	[206]
20.	Tetracycline and oxytetracycline and chlortetracycline	381 nm	456 nm	TC (1–60 μM), OTC (1–40 μM) and CTC (1–80 μM)	IFE	[207]
21.	Nimesulide	330 nm	408 nm	0–100 μM	IFE	[208]
22.	Curcumin	380 nm	467 nm	0.1–35 μM	IFE	[209]
23.	Clioquinol	360 nm	440 nm	40.0–400.0 μmol L^−1^	IFE	[211]
24.	Berberine hydrochloride	370 nm	452 nm	0.5–30.0 μmol/L	IFE	[212]
25.	Diacerein	325 nm	412 nm	2.5–17.5 μg/mL	IFE	[213]
26.	Captopril	520–550 nm	–	1–50 μM	IFE	[214]
27.	Daunorubicin and doxorubicin	–	450 nm	DAN (54.37–13594.34 nmolL^−1^) DOX (86.2–17242 nmolL^−1^)	IFE	[215]
28.	Mitoxantrone	560 nm	655 nm	0.096–30 μM	IFE	[216]
29.	Gefitinib	320 nm	410 nm	0.1–20 μg/mL	IFE	[217]
30.	Doxorubicin	491 nm	591 nm	1–30 μM	IFE	[218]
31.	Nitazoxanide	340 nm	418 nm	0.25–50.0 μM	IFE	[219]
32.	Myricetin	360 nm	436 nm	0.2–112 μM	IFE	[220]
33.	Levocetirizine and niflumic acid	319 nm	392 nm	(levocetirizine − 1.0–100 μM and niflumic acid − 0.5–100 μM)	IFE	[221]
34.	Vitamin B_12_	545 nm	595 nm	1–65 μM/70–140 μM	Internal filtration effect	[222]
35.	Metronidazole	350 nm	425 nm	0.5–22 μM	PET	[223]
36.	Thiamine	367 nm	430 nm	10–50 μM	Quenching due to Cu^2+^ ion and regain after addition of thiamine. (Turn on)	[224]
37.	Cephalosporin	300 nm	689 nm	0.2–80 μmol/L	IFE	[225]
38.	Prilocaine	400 nm	485 nm	2.3–400 nmol L^−1^	Quenching assay/Turn off the sensor	[226]
39.	Enrofloxacin	368 nm	452 nm	1–15 μg/mL	The FL was suppressed by Cu^2+^ ions and then restored by the addition of ENR, resulting in an off-on switch of the FL.	[227]
40.	Chlortetracycline	360 nm	438 nm	1–70 μM	The FL of CDs is quenched by the addition of QDs, while increases linearly with the concentration of CTC resulting in an turn off–on switch of the FL	[228]
41.	Glutathione	360 nm	430 nm	20–400 μM	The FL of CDs was quenched with the addition of Cu^2+^, by the formation of CDs-Cu^2+^ and recovered rapidly with the addition of GSH, due to the stronger interaction between GSH and CDs-Cu^2+^, resulting in an turn off–on switch of the FL.	[229]
42.	Pentachlorophenol (PCP)	360 nm	440 nm	10–300 μM	The FL of NS-CDs was significantly quenched by the addition of H_2_O_2_ and HRP and induced FL quenching. While after the addition of PCP provided a reducing environment that can protect the active group of NS-CDs from oxidation. Thus, resulting in an turn off–on switch of the FL.	[230]
43.	Ampicillin	340 nm	427 nm	6.6–200 ppm	Fluorescent quenching	[231]
44.	Nitrofurantoin	340 nm	470 nm	0–500 μM	IFE and SQ	[232]
45.	Phenobarbital	340 nm	410 nm	0.4–34.5 nmol L^−1^	Fluorescent quenching	[233]
46.	Clonazepam	340 nm	430 nm	5x10^−8^x10^−6^ M	Fluorescent quenching	[234]
47.	Acetaminophen	350 nm	–	1–80 μM	Fluorescent quenching	[235]
48.	Atorvastatin	290 nm	423 nm	0.025–50 μM	SQ	[236]
49.	Doxorubicin	360 nm	441 nm	0.5–6.5 μM	SQ	[237]
50.	Imatinib	345 nm	415 nm	1.0–15.0 mg/mL	SQ	[238]
51.	Curcumin	360 nm	440 nm	0.339–136.0 μM	SQ	[239]
52.	Tetracycline	330 nm	520 nm	0–27.27 μM	SQ	[240]
53.	Folic acid	360 nm	450 nm	10–100 μg/mL	DQ	[241]
54.	Isoniazid	270 nm	447 nm	4–140 μM	IFE and SQ	[242]
55.	Quercetin	350 nm	450 nm	0.003–80 μmol/L	IFE and SQ	[243]
56.	Tigecycline	365 nm	500 nm	0.005–20 μg/mL	IFE and SQ	[244]
57.	Tetracycline	360 nm	520 nm	0.5–40 μM	IFE and DQ	[245]
58.	Nitrotyrosine	420 nm	679 nm	20–105 μM	SQ and DQ	[246]
59.	p-benzoquinone	350 nm	–	0–25 mmol/L	DQ	[247]
60.	Methotrexate	355 nm	450 nm	0–50 μg/mL	Fluorescent quenching	[247]
61.	Doxorubicin	350 nm	–	0.5–25 μg/mL	Fluorescent quenching	[247]
62.	Chondroitin sulfate	488 nm	520 nm	0.05–2 μg/mL	Electrostatic interaction	[248]
63.	Flutamide	–	–	0.05–590 μM	–	[249]
64.	Cytochrome C	475 nm	530 nm	0.5–25 μM	IFE	[210]
65.	Zoledronic acid	340 nm	430 nm	0.1–10 μM	ZA could not quench the FL intensity of the N-CDs without Fe^3+^, once ZA was added to the N-CDs-Fe^3+^ system, the formation of a complex between ZA and Fe^3+^ ions occurred and the FL of N-CDs becomes on. (Turn on)	[250]
66.	Glucose	520 nm	582 nm	0.5–1500 μM	SQ	[251]

### 3.1 FRET-based detection mechanism

**Fig. 7:**
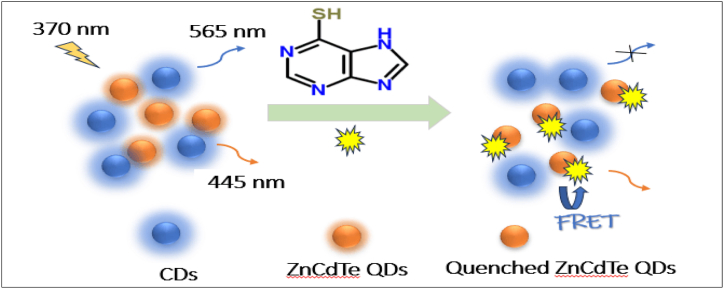
Schematic illustration of ratiometric fluorescence sensing for 6-MP.

The CDs were synthesized by adding distilled deionized water to diphosphorus pentoxide (P_2_O_5_) in glacial acetic acid, conducted in a fume hood. After cooling, a dark brown solid was obtained. The FL intensity of the CDs was pH-dependent, peaking at pH 6.0 before decreasing with higher pH levels. The CDs showed high selectivity for kaempferol (KAE), which significantly quenched their FL in the presence of other substances, indicating strong sensitivity towards KAE [191]. A method was developed to produce polyethyleneimine (PEI)-functionalized blue emissive CDs (b-CDs) using citric acid and PEI via a one-step hydrothermal process. These b-CDs were used to create a fluorescent immunoassay for simultaneously detecting chloramphenicol (CAP) and amantadine (AMD) in skinless chicken breasts. The b-CDs, along with green emissive CDs (g-CDs), featured numerous amino groups and distinct FL emission peaks. The study employed haptens of CAP and AMD as energy donors in a FRET system. Two-dimensional WS2 nanosheets (NSs) were used as energy acceptors, modified with antibodies specific to CAP and AMD. This selective antigen-antibody interaction facilitated the attachment of hapten-functionalized CDs to the WS2 NSs, resulting in FL quenching due to FRET. The immunoassay demonstrated potential for rapid detection of drug residues in food [192]. The CDs were synthesized using a self-catalysis method, with FL intensity decreasing rapidly in the first minute and stabilizing after 10 min, which was chosen for further experiments. The study assessed the impact of interfering substances like metal ions, biomolecules, and co-existing compounds, testing the method's effectiveness for real sample analysis. The results showed that the CDs were highly selective for hyperin (Hyp), with only slight changes in FL intensity at low concentrations and a significant decrease as Hyp concentration increased. The method was successfully applied to quantify Hyp in real samples, including fufangmuji granules and human serum [193]. The researchers developed a selective and sensitive fluorescent probe using zein biopolymer, functionalized with 3,5-dinitrobenzoyl chloride (DNB) to create DNB-CDs for detecting sumatriptan (SUM). The DNB-CDs were synthesized via direct pyrolysis of zein without additional reagents. Using the standard addition method for analysis, the DNB-CDs showed a strong selective response to SUM, unaffected by potential interfering substances. The study suggests that these CDs can be effectively used for determining SUM levels in human samples [194]. A one-step aqueous synthesis method was used to create Zn-doped CdTe quantum dots (ZnCdTe QDs), which were combined with B-CDs to develop a ratiometric fluorescent probe for detecting 6-mercaptopurine (6-MP). The probe exhibited different FL responses from the yellow emission of ZnCdTe QDs and the blue emission of CDs when exposed to 6-MP. After adding 6-MP, the FL intensity ratio remained stable for up to 20 min, with the probe showing the strongest response at pH 8.7. Using FRET, the FL of ZnCdTe QDs was selectively quenched, while the FL of CDs remained unaffected. The probe successfully detected 6-MP in human serum, offering a rapid method for 6-MP analysis in biological samples. Fig. 7 presents a schematic illustration of ratiometric FL sensing for 6-MP [195].

The researchers developed a sensor using N-C quantum dots (QDs) by combining a CQDs solution with sodium hydroxide, dried wheat straw powder, 2,2,6,6-tetramethylpiperidine amine, and 4,5-imidazole dicarboxylic acid. The FL intensity of the N-CQDs increased gradually at pH 7 as the gentamicin (GEN) concentration increased. The sensor's selectivity for GEN was evaluated using GEN analogs and metal ions, with metal ions causing only a slight decrease in FL intensity (around 5 %). This detection method was proven effective for accurately detecting GEN in water samples, offering a fast and simple approach with significant potential for detecting GEN residues in aqueous solutions [196]. A unique two-in-one sensor based on Au@CQDs nanocomposites (NCs) was developed for detecting gentamicin (GENTA) and kanamycin (KMC). In the synthesis, CQDs acted as reducing agents to create Au@CQDs NCs. Initially, the FL intensity of CQDs decreased due to energy transfer after the formation of Au@CQDs NCs. However, the FL intensity recovered in the presence of the antibiotics, indicating that the sensor could effectively detect both GENTA and KMC through qualitative/colorimetric and quantitative/fluorimetric methods. When tested with spiked milk and eggs, the sensor showed excellent recovery, demonstrating its potential for food safety applications [197].

### 3.2 IFE-based detection mechanism

In this study, CDs were synthesized using 4-hydroxybenzoic acid and ethylenediamine, with 4-nitrophenyl-α-D-glucopyranoside (NGP) serving as the substrate for the enzyme α-glucosidase. The enzyme catalyzed the release of 4-nitrophenol from NGP. Acarbose, a common anti-diabetes drug, was used as an inhibitor in the assays. The addition of acarbose inhibited α-glucosidase activity, leading to a recovery of FL intensity, as shown by an increase in FL when inhibitors were added. The sensor exhibited minimal interference from other substances, demonstrating its high selectivity and applicability for detecting α-glucosidase activity [198]. CDs were synthesized using diammonium hydrogen citrate and PEG-400 via a microwave-assisted method. The resulting CDs were evaluated for cytotoxicity and cell imaging, with their FL intensity showing a pH-dependent behavior. The study also investigated the FL-quenching effects of various metal ions, finding that Fe^3+^ had the most significant impact. This quenching was attributed to energy transfer processes within the excited CDs. Additionally, the CDs were successfully used for cellular imaging in BGC-823 and CT26.WT cells, showcasing their potential as optical nanoprobes for cell imaging applications due to their high FL brightness [199]. Mg-doped carbon dots (Mg-CDs) were synthesized using a simple, one-pot microwave-assisted method by dissolving citric acid in a magnesium chloride (MgCl_2_) solution and mixing it with ethylenediamine. The resulting Mg-CDs nanoprobes (Mg-CDs-0.1) demonstrated high selectivity and sensitivity for detecting hematin due to the strong IFE between Mg-CDs and hematin. Specificity tests revealed that Mg-CDs-0.1 showed a significant FL intensity reduction only in the presence of hematin, with no impact from other molecules or metal ions. Similar results were observed with Mg-CDs-0.5, confirming their high reproducibility and selectivity for detecting hematin in human red cell hemolysate [200].

**Fig. 8:**
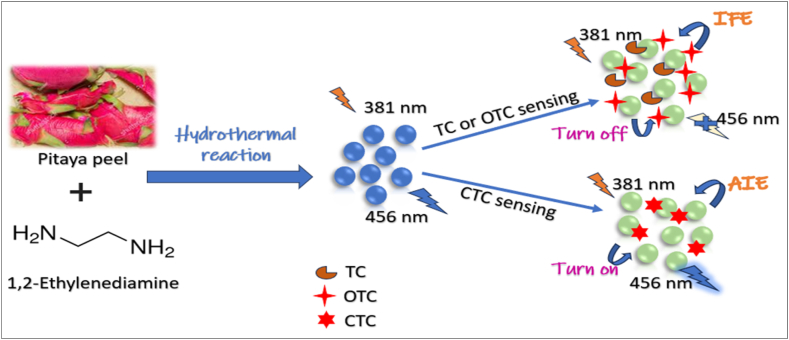
Schematic illustration of synthetic process of N-CDs and application as a multifunctional nano-sensor for sensing of TC, OTC, and CTC.

Carbon dots (CDs) were synthesized from environmentally friendly pomegranate juice using a simple hydrothermal method and tested as sensitive indicators for detecting cefixime (CEF). The detection method relies on the interaction between palladium ions (Pd (II)) and CEF in an acidic buffer (pH 4). The excitation spectra of the synthesized CDs and the Pd (II)-CEF complex were found to overlap, leading to a decrease in FL intensity when both CEF and Pd (II) were present, enabling quantitative detection of CEF. The technique showed minimal interference from other ions and biomolecules. The method was successfully applied to analyze urine samples from a healthy individual and pharmaceutical formulations, demonstrating its potential for rapid CEF detection in real samples [201]. A rapid and efficient microwave-assisted method was used to synthesize glycerol-urea CDs (GUCDs). The ability of GUCDs to detect antibiotics in urine samples was tested by adding various compounds, including antibiotics, to GUCDs solutions. The FL signal of GUCDs remained mostly unaffected by the presence of other molecules, except for tetracycline (TC) antibiotics (TC, doxycycline, and oxytetracycline (OTC)), which significantly altered the responses. The FL intensity of GUCDs was pH-dependent, with the highest emission observed at pH below 4.0. This pH was chosen to maximize the FL signal and prevent TC degradation. As a result, a simple, low-cost method based on the reduction of GUCDs FL was developed to detect TC in urine samples, indicating the high selectivity of GUCDs for TC antibiotics [202]. In a study focused on the development of a fast FL sensor, CDs were synthesized using hawthorn, and the sensor was designed to detect chlortetracycline (CTC) in pork samples. The results showed that substances like CAP, sulfanilamide, and florfenicol did not interfere with the sensor's reaction system. However, TC antibiotics (such as TC, doxycycline, and OTC) did affect the FL response, with TC having the strongest impact. This highlighted that the N-CDs were particularly selective for detecting TC antibiotics, making them useful for detecting harmful substances in food [203]. The europium doped carbon dot (Eu-CDs) were synthesized using citric acid, melamine, and Europium (III) Nitrate Hexahydrate (Eu(NO₃)₃·6H₂O) in a one-pot hydrothermal method, with Eu serving as a TC-binding site and providing FL. Formaldehyde was added as a passivating agent to modify the nitrogen's chemical state in the Eu-CDs. The study found that the maximum FL intensity ratio for both TC and Al³⁺ occurred at pH 8.0, which was identified as the optimal pH. The Eu-CDs exhibited stable FL and color, unaffected by biological ions or other antibiotics. Notably, only TC caused a significant FL enhancement at 620 nm and a color shift from blue to red, demonstrating the Eu-CDs' high selectivity for TC detection [204]. In a separate study, B-CDs were synthesized with the help of salicylic acid, melamine, and distilled water, incorporating zinc nitrate hexahydrate and 2-methylimidazole. The CDs were then modified into a hierarchical mesoporous zeolitic imidazolate framework-8 (HZIF-8) using hydrogel as a template. The specificity of the CDs@HZIF-8 system for TC was evaluated by introducing various potential interfering substances, such as metal ions, amino acids, vitamins, and antibiotics. The results showed that the sensor maintained excellent selectivity and anti-interference capacity for TC, confirming its robustness for real-world applications [205]. A novel FL sensor was developed using nitrogen-doped carbon dots (N-CDs) embedded in zinc-based metal-organic frameworks and molecularly imprinted polymer (ZIF-8&N-CDs@MIP). This sensor, optimized at pH 7, demonstrated high sensitivity and selectivity for TC, with minimal interference from its structural analogs, such as CAP, OTC, and CTC. The sensor showed excellent selectivity, improved sensitivity, and fast response times for detecting TC [206]. N-CDs were synthesized using pitaya peel and 1,2-ethylenediamine via a hydrothermal method. The N-CDs exhibited a “turn-off” FL response when interacting with TC and OTC, while showing a “turn-on” FL response with CTC, making them highly sensitive for CTC detection. Selectivity tests demonstrated that the FL intensity of N-CDs was minimally affected by other potential interfering substances, including metal ions, antibiotics, amino acids, and anions. The sensor proved to be a versatile, dual-mode platform for detecting TC with high sensitivity and adaptability. For example, the schematic illustration of the synthetic process of N-CDs and their application as a multifunctional nano-sensor for sensing TC, OTC, and CTC is shown in Fig. 8 [207].

In this study, CDs were synthesized using trimesic acid and 4-amino acetanilide hydrochloride through a one-step hydrothermal process. This method involved combining glucose solutions of varying concentrations with glucose oxidase (GOx) in a Britton-Robinson buffer at 37 °C for 40 min to produce hydrogen peroxide (H_2_O_2_). When CDs were added to the mixture, their FL was quenched by H_2_O_2_, with the quenching intensity being directly proportional to the H_2_O_2_ concentration, enabling glucose detection. The method showed good sensitivity and selectivity when tested with biological samples, including human serum and urine from diabetic and healthy individuals. Additionally, B, N-doped CDs were synthesized using ammonium citrate and bis(pinacolato)diboron via a hydrothermal method. Interference studies revealed that nimesulide significantly quenched the FL, while other substances had little to no effect. This demonstrated the high sensitivity and selectivity of the sensor. Furthermore, the B, N-CDs proved useful for fluorescent staining and anti-counterfeit applications. The method was successfully applied to pharmaceutical samples for detecting Nimesulide [208].

N and Cl-functionalized CDs (N, Cl-CDs) were synthesized quickly and environmentally using glucose, ethylenediamine, and HCl. The FL intensity of the N, Cl-CDs was rapidly quenched by curcumin (Cur), indicating its effective interaction with the CDs. Selectivity tests showed that Cur was the only substance among the investigated compounds that significantly reduced the FL intensity, while other potential interfering substances had little to no impact. This demonstrates that N, Cl-CDs exhibit high selectivity for Cur and are unaffected by interference from other drugs, making them a promising tool for Cur detection [209]. A study developed a label-free probe for detecting cytochrome *c* (Cyt C) using nitrogen and fluorine co-doped carbon dots (N, F-CDs), which were easily synthesized through a solvothermal method using 3,4-difluorophenylhydrazine as a precursor. The results demonstrated that the N, F-CDs probe exhibited excellent anti-interference properties, making it a promising candidate for detecting Cyt C. Additionally, the probe showed potential as a temperature sensor with higher sensitivity, suggesting its broad application in biosensing [210].

In this study, CDs were synthesized using ammonium citrate and ammonium thiocyanate through a hydrothermal process. The addition of Cu^2^⁺ initially increased the FL intensity of the CDs. However, when clioquinol (CQ) was introduced, its high affinity for Cu^2^⁺ displaced Cu^2^⁺ from the CDs surface, leading to FL quenching due to IFE. The study examined the effects of pH, ionic strength, and UV exposure time on the FL intensity. The difference in FL intensity between the CDs + Cu^2^⁺ system and the CDs + Cu^2^⁺ + CQ system was greatest at pH 8.0. Cu^2^⁺ adsorbed on the CDs surface, stabilizing them and preventing non-radiative recombination, which increased FL intensity. A new UV absorption peak confirmed the formation of the Cu^2^⁺-CQ complex. Interference studies showed that CQ could significantly quench the FL of CDs, while other metal ions had negligible effects. This system was proposed as a fluorescent sensor for detecting CQ [211].

Silicon-doped carbon quantum dots (Si-CQDs) were synthesized through a straightforward one-pot hydrothermal method using 3-aminopropyltrimethoxysilane and H_2_SO_4_. The pH had no significant effect on the FL behavior of the Si-CQDs. The study also examined how the FL intensity of Si-CQDs was influenced by the addition of various metal ions, amino acids, saccharides, and other substances that could be found in urine samples. Among these, only berberine hydrochloride (BH) caused a significant reduction in FL intensity, while other drugs had minimal effects. These results demonstrate that the developed sensor has high sensitivity and selectivity for BH detection [212].

Chitosan was utilized as a C and N source in the carbonization process to develop a fluorescent sensor. The FL of the resulting probe was effectively quenched by diacerein (DIA), producing rapid and stable FL responses. The sensing system demonstrated high precision and accuracy, allowing for the selective detection of DIA in its tablet dosage form, even in the presence of co-formulated medications [213]. Rhodamine B was dissolved in ultrapure water and used in a solvothermal process to synthesize CDs. Various chemicals, including sugars (glucose, sucrose), inorganic ions (K^+^, Na^+^, Ca^2+^, Cl^−^, NO_3_^−^), amino acids (histidine, alanine, threonine, phenylalanine, arginine), and medications (paracetamol, artemisinin), were tested for their impact on the sensor. When these substances were added individually without captopril (CP), there was no significant change in the FL and absorbance signals. However, the addition of CP led to a substantial enhancement in both FL and absorbance signals. These results demonstrated the high selectivity and robustness of the CP sensor, which was able to tolerate interference from coexisting chemicals [214].

Using a one-pot green synthesis method, a green bell was chosen to produce CDs due to its high-quality contents, including carotenoids, ascorbic acid, carbohydrates, and other carbonaceous organic compounds. To create MSA-CdTe quantum dots (QDs), cadmium chloride (CdCl_2_), mercaptosuccinic acid (MSA), sodium tellurite (Na_2_TeO_3_), and a borate-acetic acid buffer solution were combined. Then, Si@CdTe QDs were synthesized using MSA-CdTe QDs, 3-Aminopropyl triethoxysilane (APTES), and ethanol. The ideal pH for detecting doxorubicin (DOX) and daunorubicin (DAN) was found to be pH 7. Sodium chloride (NaCl) did not cause significant changes in the signal intensity, which was used to assess the impact of potential interferents like glutamine, fructose, and glucose in biological samples. These results demonstrated that the ratiometric fluorescence (RF) sensors were highly effective in detecting both DAN and DOX [215].

Red-emission carbon dots (R-CDs) were synthesized using a solvothermal process involving formamide, N, N-dimethylformamide, and citric acid. The optimal pH for the reaction system was found to be 6.0. When used as a fluorescent probe for detecting mitoxantrone (MITX), R-CDs exhibited excellent selectivity and resistance to interference, as the presence of interference compounds had little to no effect on either the R-CDs or the R-CDs-MITX system's FL. The developed label-free fluorescent nanoprobe proved to be highly effective for the rapid and accurate detection of MITX in human serum samples [216]. The green tea leaf residue was used as the carbon source for synthesizing carbon dots (named as T-CDs) employing a combination of high-temperature pyrolysis and oxidation with concentrated H_2_SO_4_. The FL intensity of the T-CDs was found to decrease as the concentration of gefitinib increased, with the FL quenching effect being proportional to the drug concentration. Selectivity tests revealed that no other substances at a concentration of 20 μg/mL significantly affected the FL of T-CDs, except for gefitinib, indicating the high specificity of T-CDs for gefitinib detection. The method utilized the IFE of gefitinib to quench the FL of T-CDs, and it was successfully applied to detect gefitinib in urine samples [217]. A study developed a FL probe for detecting DOX using plum-based carbon quantum dots (PCQDs) synthesized via a simple bottom-up method. This ratiometric FL probe was tested on urine and serum samples, which may contain interfering substances. The results showed that, apart from DOX, the absorption peaks of interfering chemicals did not overlap with the emission peak of the PCQDs. Only DOX significantly increased the I591/I491 ratio of the PCQDs, indicating a strong response. The probe exhibited high selectivity for DOX over other drugs, such as cytarabine, cytoxan, 5-fluorouracil, and methotrexate, which had no effect on the I591/I491 readings. This demonstrated that the presence of other substances did not interfere with the detection of DOX, making the probe a reliable and selective tool for DOX detection in complex samples [218].

Plant-based sulfur and nitrogen self-co-doped carbon quantum dots (S, N-CQDs) were synthesized in an environmentally friendly, cost-effective, and rapid one-pot process using onion and cabbage juices and distilled water. The optimal pH for the procedure was determined to be 8.0. The applicability of the synthesized S, N-CQDs for FL sensing of nitazoxanide and hemoglobin (Hb) was evaluated by testing their response to various potential interfering substances, including glycine, glucose, sucrose, citric acid, sodium benzoate, sodium citrate, urea, lysine, nicotinamide, glutathione, and various ions (Ca^2^⁺, K⁺, Na⁺, Mg^2^⁺, Cl⁻, SO₄^2^⁻). The results demonstrated that these green, safe, affordable, and sustainable S, N-CQDs have significant potential for use in pharmaceutical and biological applications [219].

**Fig. 9:**
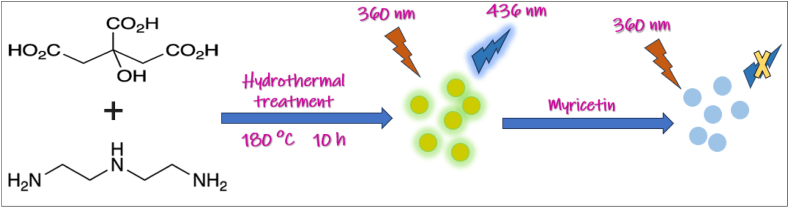
Schematic diagram about the preparation process of N-CDs and detection principle for myricetin.

N-CDs were synthesized using diethylenetriamine and citric acid through a hydrothermal process. The FL intensity of the sensor decreased linearly with an increase in myricetin concentration. The sensor showed high selectivity for myricetin, as its signal intensity was unaffected by various structural analogs such as warfarin, KAE, and luteolin, among others. Additionally, the sensor exhibited strong anti-interference properties, with minimal impact from potential interfering ions or compounds, ensuring reliable detection of myricetin. The schematic diagram of the preparation process of N-CDs and the detection principle for myricetin is shown in Fig. 9 [220].

Water-soluble carbon dots (SD-CDs) were synthesized using a microwave-assisted approach with 5-sulpha anthranilic acid (SAA) and 1,5-diphenylycarbazide (DPC) as precursors. The selectivity of SD-CDs towards levocetirizine and niflumic acid was investigated in the presence of various metal ions (Na^+^, Ni^2+^, Mn^2+^, Cd^2+^, K^+^, Ca^2+^), anions (Br^−^, SO_4_^2−^, Cl^−^, NO_3_^−^), and drugs. The results showed that the FL intensity of SD-CDs was enhanced exclusively by levocetirizine, even in the presence of other interfering substances. On the other hand, niflumic acid caused a significant FL quenching when mixed with other chemicals, demonstrating a high selectivity of SD-CDs for these two compounds. The addition of metal ions, anions, and other drugs did not result in FL enhancement or quenching, confirming the robustness and specificity of SD-CDs. These findings indicate that SD-CDs could serve as a reliable analytical platform for detecting levocetirizine and niflumic acid in real-world samples [221].

Orange-emission fluorescent multifunctional carbon dots (O-CDs) were synthesized using safranine T and ethanol through a one-step hydrothermal process. These O-CDs exhibit excitation-independent FL properties, making them suitable for applications in biosensing, cellular labeling, and photovoltaic materials. When tested for selectivity, O-CDs showed significant FL quenching only in the presence of vitamin B_12_ (VB_12_), indicating their high selectivity for VB_12_ analysis. The O-CDs also demonstrated a longer FL emission duration compared to other fluorescent techniques for VB_12_ analysis, making them highly effective for detecting VB_12_ in biological samples. The mechanism of FL quenching was attributed to IFE, confirmed through UV–Vis absorption spectra and lifetime measurements, showing that VB_12_ absorbed the excitation spectrum of O-CDs, leading to FL quenching. This specificity was further validated as the FL did not change in the presence of amino acids or other vitamins [222].

### 3.3 PET-based detection mechanism

In a study using a high-pressure microwave method, biomass CDs were synthesized from longan peels. To enhance the selectivity and sensitivity of the fluorescent probes, CDs were integrated with molecularly imprinted polymers (MIPs) and restricted access materials (RAM-MIPs), resulting in the creation of CDs@RAM-MIPs. The results showed that CDs@RAM-MIPs exhibited excellent selectivity for metronidazole (MNZ), with no significant interference from other drugs. The unique recognition cavities of the CDs@RAM-MIPs selectively bind MNZ, allowing for precise detection of the target molecule. The method's applicability was also evaluated by measuring MNZ levels in serum samples, demonstrating its potential for accurate detection in complex biological samples [223].

### 3.4 On, off, and off-on based detection mechanism

Fluorescent CDs were synthesized by a microwave-assisted method using a mixture of coconut water and ethanol (1:1 v/v). The resulting CDs displayed luminescent properties, which were influenced by the reaction temperature and microwave exposure time during the synthesis. The study showed that pure coconut water itself did not exhibit any FL, but the CDs produced during the microwave reaction displayed strong FL. These CDs were then used as a sensing platform for thiamine detection. The FL of the CDs was quenched by the addition of Cu^2+^ ions. However, when thiamine was added to the mixture of CDs and Cu^2+^, the FL intensity was restored to its original value. This restoration of FL intensity was directly proportional to the concentration of thiamine, allowing for the quantification of thiamine in real samples such as blood and urine. The method proved highly selective for thiamine, with minimal interference from other potentially coexisting molecules and ions. This makes the CDs a useful tool for thiamine analysis in biological samples, demonstrating a practical and sensitive approach to thiamine detection [224].

In a separate study, N-CDs were synthesized using a hydrothermal method to detect zoledronic acid (ZA) in blood serum. The N-CDs exhibited a FL intensity change when ZA was added. Specifically, the FL was quenched when Fe^3+^ ions were present, but it was restored upon the introduction of ZA, forming a complex with Fe^3+^. The optimal pH for this FL sensing system was found to be 4.0, using an acetate buffer. This method showed high selectivity for ZA, as there were no significant FL changes when other molecules or ions, even in excess, were added. The study also compared the FL response of ZA to other substances like fludarabine (FLD), a chemotherapy drug, and found that FLD did not interfere with the sensing process. This demonstrates that the N-CDs-Fe^3+^ complex can be effectively used for selective and sensitive detection of ZA in blood serum, highlighting the potential of N-CDs for chemical sensing and medical applications [225].

Saffron was used as a precursor to synthesize CDs through a hydrothermal method, offering a novel and cost-effective approach. The fluorescent properties of these CDs enabled the sensitive detection of prilocaine in human plasma. Additionally, the synthesized CDs were applied for bioimaging, specifically for imaging olfactory mucosa cells and bone marrow cells. The results demonstrated excellent imaging capabilities, suggesting that these saffron-derived CDs are promising candidates for bioimaging applications, particularly in cancer cell imaging. This highlights their potential for medical and diagnostic use [226].

The researchers synthesized N-CDs using DL-malic acid and glycine. Initially, adding enrofloxacin (ENR) to the N-CDs caused no change in the FL intensity or shape, indicating no interaction between the two. However, the introduction of Cu^2+^ restored the FL of the N-CDs, suggesting that Cu^2+^ interacts with both ENR and the N-CDs. Additionally, the concentration of N-CDs in the solution increased with the addition of Cu^2+^, likely due to the aggregation of some N-CDs or the formation of larger complexes between Cu^2+^ and ENR. This approach was shown to selectively detect ENR in tap and river water samples, demonstrating its potential for environmental monitoring [227].

In a study, the FL intensity of CDs increased with rising concentrations of CTC, with a blue shift in the emission peak. This sensor showed excellent selectivity for CTC over other TC antibiotics, structurally similar drugs, various ions, and naturally occurring amino acids. The sensor was also effective in detecting CTC in milk and tap water, indicating its application in food safety and environmental testing [228].

**Fig. 10:**
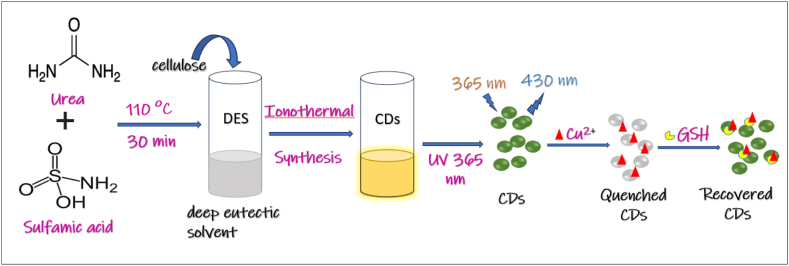
Schematic preparation of N, S-CDs and its detection for Cu^2+^ and GSH.

Moreover, N, S-CDs were synthesized using a one-pot ionothermal method with deep eutectic solvent and microcrystalline cellulose (MCC) as solvents and dopants. These N, S-CDs were used to detect glutathione (GSH), where the FL quenching caused by the formation of a CD-Cu^2+^ complex was reversed by adding GSH. The interaction between GSH and Cu^2+^ restored the FL, and as GSH concentration increased, the FL became more intense. This work demonstrated that GSH is selective for the recovery of CD FL, and the study also evaluated the impact of interfering substances like amino acids and small reducing molecules. The findings suggest broad applications for biomass-derived carbon dots in selective FL sensing. The schematic illustration of the preparation of N, S-CDs and their detection capabilities for Cu^2^⁺, and GSH is shown in Fig. 10 [229].

Food-derived crawfish shells were utilized as green precursors to synthesize N, S-CDs via a hydrothermal method. The FL of the N, S-CDs was significantly quenched upon the addition of H_2_O_2_ and horseradish peroxidase (HRP). This quenching occurred because the hydroxyl radicals generated by the reaction between H_2_O_2_ and HRP oxidized the surface groups (N, sulfur, and oxygen) of the N, S-CDs, altering their surface states and causing FL suppression. However, when pentachlorophenol (PCP) was introduced to the NSCDs@HRP/H_2_O_2_ system, the FL was restored. PCP provided a reducing environment that prevented the oxidation of the active groups on the N, S-CDs, thus reactivating the FL. This fluorescent sensing method proved highly effective for detecting PCP in real samples, highlighting the potential of this approach for environmental and analytical applications [230].

### 3.5 Quenching-based detection mechanism

In one study, CDs were synthesized through hydrothermal treatment using glucose, ampicillin, 2-phenyl glycine, and (+)-6-amino penicillanic acid. The β-lactam subunit within ampicillin, with its N and S atoms, was identified as the key factor contributing to the unique absorption and emission properties of the resulting CDs. Unlike a glucose solution without hydrothermal treatment, the product solutions containing varying amounts of ampicillin exhibited distinct FL, showcasing the significant role of ampicillin in the FL properties of the CDs [231]. In a study involving co-doped CNDs containing N and phosphorus (P), the synthesis was achieved using FA through a single-step solvothermal method. The interference study showed that the FL intensity of the CNDs remained almost unchanged when exposed to common excipients like lactose, L-ascorbic acid, talc, magnesium tartrate, and starch, suggesting that these substances did not interfere with the analytical method. This indicates the reliability of the CND-based sensor. Furthermore, in vivo studies revealed that the CNDs had no significant cytotoxic, hematological, or biochemical effects on animals, supporting their biocompatibility [232].

In another study, Cedrus was used as a green source for producing CDs through a hydrothermal method. These CDs were then modified with MIPs using reverse micro-emulsion technology, resulting in MIPs-Green Source CDs (MIPs-GSCDs). The sensor was optimized in terms of pH, temperature, and response time. The MIPs-GSCDs exhibited high sensitivity and selectivity for phenobarbital in human blood plasma, demonstrating the practical application of this sensor for real sample analysis [233].

A separate study used citric acid and ammonia to synthesize CDs through pyrolysis at high temperatures. These CDs were tested for their selectivity in plasma and pharmaceutical formulations, particularly in the presence of potential interfering substances. While most compounds had little effect on the FL of CDs, clonazepam caused a significant quenching of FL, likely due to interactions between its nitro group and the surface functional groups of the CDs. This high selectivity allows for the precise detection of clonazepam in low concentrations, making the sensor valuable for monitoring trace amounts in pharmaceutical and plasma samples [234].

Additionally, red-emitting copper nanoclusters (CuNCs) were synthesized using DNA as a template and combined with blue-emitting CDs to form a self-assembled complex, DNA-CuNC/CDs. This complex was tested as a ratiometric FL nanoplatform for detecting arginine (Arg) and acetaminophen (AP) in human serum samples. Upon the addition of AP, the FL of the complex shifted from purplish-red to blue, and the FL of the CDs was restored, demonstrating the ability to detect AP. The system showed excellent selectivity and sensitivity, with minimal interference from other substances. This ratiometric FL nanoplatform also highlighted the potential for building “INHIBIT” logic gates at the molecular level, offering exciting prospects for early disease diagnosis and real-time clinical monitoring [235].

A microwave-assisted method was used to synthesize CDs from glucose and deep eutectic solvents (DESs), composed of choline chloride and urea mixed with deionized water. The CDs were tested for FL properties by mixing them with various ions and molecules at a concentration of 3 μM. The FL intensity remained mostly unaffected by most species, except for atorvastatin, which caused significant quenching, indicating the sensor's high selectivity for atorvastatin. FL quenching occurred quickly at room temperature after adding atorvastatin. The FL intensity was pH-dependent, increasing between pH 3–8, with a peak at pH 7.4, which was chosen as the optimum pH for the sensor. The FL intensity decreased significantly with increasing atorvastatin concentration, demonstrating the CDs sensitivity for atorvastatin detection. Nitrogen and chloride-doped carbon dots (N/Cl-CDs) were synthesized using the same method to enhance the sensor's selectivity and sensitivity. These N/Cl-CDs were successfully used as a fluorescent nanosensor to detect atorvastatin in blood, showcasing their potential for monitoring atorvastatin in biological samples [236].

**Fig. 11:**
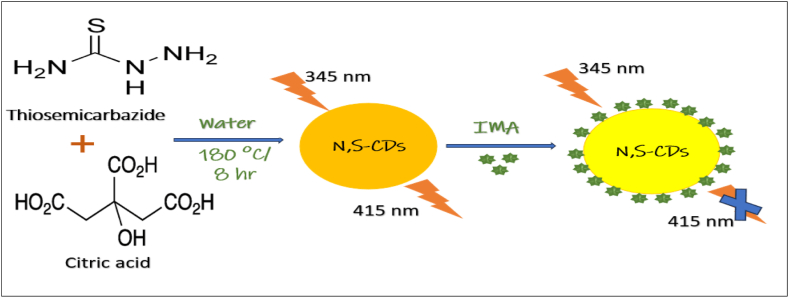
Outline of the synthesis process and applications of N,S-CQDs.

Hydrothermal synthesis was used to create nitrogen and phosphorus co-doped carbon dots (N, P-CDs) with intense blue FL using alanine and diammonium phosphate. The FL intensity remained stable with an increase in pH until DOX was introduced, which caused a significant decrease in the FL intensity, indicating that the N, P-CDs were sensitive to DOX. The FL intensity reached its peak at pH 7.0, and with increasing concentrations of DOX, the FL intensity gradually reduced. When tested against various common metal cations and biomolecules, only DOX caused a marked “turn-off” effect, demonstrating the high selectivity of the N, P-CDs for DOX detection [237]. Thiosemicarbazide and citric acid were utilized in a one-step hydrothermal method to synthesize nitrogen and sulfur-doped carbon quantum dots (N, S-CQDs). These N, S-CQDs demonstrated strong FL and were used for the detection of imatinib (IMA) in pharmaceutical and biological samples. As the concentration of IMA increased, the FL intensity of the N, S-CQDs decreased, providing a simple and sensitive approach for IMA detection, with the added benefits of low cost and ease of use. The systematic detection process of imatinib is depicted in Fig. 11 [238].

Similarly, a fluorescent sensor for Cur detection was developed based on N, S-CQDs and MIPs. The CQDs with blue FL were synthesized using citric acid and o-phenylenediamine. The sensor, composed of N, S-CQD@MIP, showed higher quenching efficiency for Cur compared to the N, S-CQD@NIP composite, highlighting its superior sensitivity. Even when the concentration of interfering drugs was ten times higher than Cur, the FL quenching efficiency remained largely unaffected, demonstrating excellent selectivity for Cur. Further tests with various ions and proteins confirmed that the sensor was highly selective and specific for Cur detection [239]. Highly luminous CDs were synthesized from wild lemon leaves using one-step microwave pyrolysis. The selectivity of the CDs was tested by introducing a variety of foreign substances, including biomolecules, metal ions, and antibiotics. The CDs demonstrated exceptional selectivity for TC detection, as they remained highly fluorescent even in the presence of various interferents. This biocompatible, label-free nanoprobe was successfully used to detect TC in environmental water samples, showing good recovery rates and promising potential for environmental monitoring [240].

**Fig. 12:**
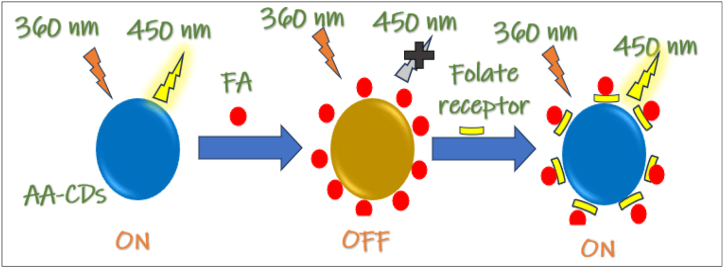
Schematic illustration for the detection of folic acid.

Researchers synthesized Aconitic acid-based carbon dots (AA-CDs) using aconitic acid (AA) and 1,2-ethylenediamine via a hydrothermal method. They found that the addition of FA could effectively quench the intrinsic FL of AA-CDs without requiring additional surface modifications or passivation. The AA-CDs were successfully used for detecting FA in food and pharmaceutical samples. Additionally, the researchers demonstrated the potential of FA-AA-CDs for targeted imaging, as they were able to distinguish cancer cells (HeLa, SMMC-7721, and A549) based on varying levels of folate receptor's (FRs) expression. This showed the feasibility of using turn-on FL for imaging cancer cells with overexpressed FRs indicating a promising application for cancer diagnostics and imaging. Fig. 12 presents a schematic illustration of ratiometric FL sensing for FA [241].

**Fig. 13:**
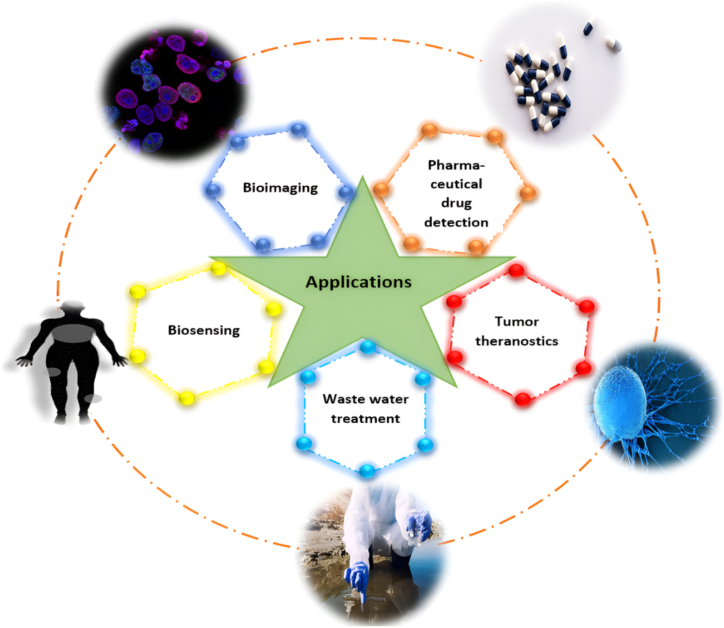
Various applications of CDs.

A new type of fluorescent CDs was synthesized by pyrolyzing folic acid. These fluorescent CDs exhibit excitation-independent FL, allowing for rapid and sensitive detection of isoniazid through a combination of IFE and SQ effects. This makes them promising for drug testing applications, potentially offering new methods and technologies for detecting pharmaceutical compounds [242]. Another study focused on the synthesis of fluorescent nitrogen-doped carbon quantum dots (N-CQDs) using a hydrothermal process involving natural osmanthus fragrans, without the use of harmful substances or surface chemical modifications. In tests for potential interference, the FL intensity of N-CQDs remained stable when mixed with various metal cations, indicating that they are highly selective for quercetin (QT) sensing. These N-CQDs can serve as a reversible, sensitive platform for detecting QT, making them suitable for clinical diagnostics and other biomedical applications [243].

Additionally, Fluorine, Nitrogen-doped carbon dots (F, N-CDs) were synthesized using tetrafluoroterephthalic acid and tetraethylenepentamine. These F, N-CDs exhibited a rapid decrease in FL intensity as the concentration of tigecycline (TIGE) increased. The FL quenching effect was highly specific, as other potential interferences showed minimal impact. The method demonstrated strong anti-interference properties, making it suitable for real-time drug monitoring. The approach was successfully applied for detecting labeled TIGE and TIGE injections in human plasma, highlighting its potential for precise and reliable drug monitoring in clinical settings [244].

A sensor was developed by coupling ovalbumin (OVA) and 3-aminophenyl boronic acid (3-APBA) with CDs through the specific interaction of cis-diol bonds in glycoproteins and borate groups on the CDs surface. This CDs-functionalized OVA acted as both a stabilizing and reducing agent during the synthesis of gold nanoclusters (AuNCs), forming a CDs-AuNCs nanocomposite. The sensor demonstrated the highest ratiometric FL efficiency at pH 8.5, producing a bright yellow color. The sensor was tested for TC detection, showing high selectivity and minimal interference from other antibiotics, amino acids, and potential contaminants. This triple-emission sensor is highly selective for tetracyclines (TC's) and has potential for practical applications in antibiotic detection [245]. In a single step, CDs were synthesized using GSH and formamide via a microwave-mediated process. Interference studies demonstrated that the CDs were highly selective for detecting nitrotyrosine (nTyr), even in the presence of other biologically significant compounds like tyrosine (Tyr) and phosphotyrosine (pTyr). Without nTyr, the FL emission of the CDs was only slightly reduced (by 10 %) in the presence of these interferents, indicating that the CDs were specifically designed for nTyr detection with minimal interference [246].

The CDs were synthesized using a hydrothermal method with citric acid as the carbon source and ethylenediamine as the co-reactant. These CDs were employed as probes to detect dopamine (DA) and DOX in human serum solutions. In the serum, DA effectively quenched the FL of the CDs, establishing a strong linear correlation. Similarly, DOX was also detected with a good linear relationship in the serum solution. The results indicate that CDs are highly suitable for detecting DA and quinone-based drugs, such as DOX, in human serum due to their excellent stability, eco-friendliness, and anti-interference properties. The method was also successfully applied to quantitatively identify DOX and MITX, both of which share a typical quinone structure. This approach provides a solid foundation for further biological applications, offering reliable and efficient detection capabilities in clinical and pharmaceutical settings [247].

Fluorescent CDs were synthesized using citric acid and L-glutathione to create a platform for detecting chondroitin sulfate (CS). The positively charged N-doped CDs (P-NCDs) were combined with FAM-labeled random-sequence single-stranded DNA (F-ssDNA), creating a sensitive and simple ratiometric homogeneous assay through competitive electrostatic interactions. The process of CS detection was confirmed by measuring the zeta potential, which decreased due to the electrostatic interaction between negatively charged CS and P-NCDs/F-ssDNA. The zeta potential decreased significantly when CS was added, showing a stronger interaction between P-NCDs and CS (−12.5 mV) compared to P-NCDs and F-ssDNA. Further validation was achieved by comparing the zeta potential of P-NCDs with CS (−4.5 mV) to that of their mixture (−19.75 mV). The system was successfully applied to detect CS in actual joint fluid, demonstrating its potential for clinical use. This study highlights new insights into the development of fluorescent nanomaterials and DNA for biosensing applications [248].

CDs were synthesized using CO(NO_3_)_2_.6H_2_O and cetrimonium bromide (CTAB), followed by dispersion with multi-walled carbon nanotubes (MWCNTs) via ultrasonication. The FL intensity of the CDs was found to be pH-dependent, with the highest redox peak current observed at pH 7.0 for the drug molecules flutamide (FLU) and nitrofurantoin (NF). The sensor was evaluated for practical use in human urine samples, showing its capability for the simultaneous detection of these drug molecules. This study demonstrates a straightforward method for synthesizing a hybrid nanocomposite and highlights its potential for drug detection applications [249].

## 4 Current challenges and future prospects

Many pharmaceutical drugs have been detected using HPLC, HPTLC, UV–Vis, and Fourier transform infrared spectroscopy (FT-IR) to date. Analytical experiments (sampling, sample preparation, instrumental analysis, data processing, and data interpretation) present distinct problems in achieving the goal of enhancing the existing state of pharmaceutical analysis and, more broadly, functionalization. To overcome these challenges CDs can be used for detection just by converting the non-fluorescent substances into fluorescent substances and sensing through a different mechanism. Pharmaceutical residues of antibiotics, nonsteroidal anti-inflammatory drugs (NSAIDs), lipid-regulators, hormones, β-blocker, anticonvulsants, steroids, opiate drugs, antidepressants, and anti-diabetics in the environment have recently been discovered as a hazard, with their presence in the aquatic environment being particularly critical [252]. Experiments conducted in both laboratory and natural settings have revealed that oral contraceptives are leaving traces in water bodies and causing the feminization of fish and amphibians, while psychiatric drugs are leaving residues that alter fish behavior. Furthermore, the excessive use of antibiotics has led to a rapid rise in antimicrobial resistance, which has become a major global health crisis [253]. Previous studies have found that common antidepressant medications are accumulated in the brain tissue of fish that inhabit water downstream from wastewater treatment plants (WWTPs) [254]. Numerous techniques have been proposed for eliminating antibiotics, including advanced oxidation, reverse osmosis, membrane filtration, electrochemical methods, and biological treatments. However, most of these methods are expensive, produce by-products, or are not as efficient. Removal of antibiotics by using various adsorbents (sawdust, activated carbon, NPs, sludge biochar) has been reported. Antibiotics such as gatifloxacin have been removed by more than 90 % using NPs and 68.5 % and 64 % of ciprofloxacin by using kandira stone and sawdust as an adsorbent [255]. The Fenton oxidation process combined with ultrafiltration (UF), sand filtration, reverse osmosis (RO), and nanofiltration (NF) resulted in recovery above 90 %. In another study, the electro-Fenton process was used to remove diclofenac sodium where about 80 % drug removal was observed [256]. Recently, various techniques have been developed to remove antibiotics and antibiotic resistance genes. These techniques include the use of Fe_3_O_4_/red mud NPs, 3D hierarchical porous-structured biochar aerogels, calcined layered double hydroxides, co-doped UiO-66 NPs, Cu@TiO_2_ hybrids, bioelectrochemical systems, and aerobic granulation process. The results of studies have shown that most of these methods are effective in removing antibiotic residues and antibiotic resistance genes, with removal rates ranging from 85 % to 95 % [257]. A recent study found that a mix of domestic and livestock sewage in rural wastewater contained five steroid hormones [androsta-1,4-diene-3,17-dione (ADD), 4-androstene-3,17-dione, 19-norethindrone (19-NTD), testosterone (T), and progesterone ] and four biocides [N, N-diethyl-3-methylbenzamide (DEET), triclosan (TCS), carbendazim (CBD), and methylparaben (MP)]. However, the results showed that an integrated constructed wetland (ICW) was able to significantly reduce the levels of the detected hormones and biocides, with a reduction rate of 97.4 ± 0.09 % and 92.4 ± 0.54 %, respectively [258].

Due to the physical and chemical complexity of the NSAIDs compounds, there is no single method that is sufficiently effective against all types of contaminants [259]. Evidence reveals that pharmaceutical chemicals, physicochemical properties, and structural complexity make them difficult to remove completely in traditional WWTPs, highlighting their unintentional persistence in the environment. There is a toxic effect of pharmaceutical compounds on the human body at low concentrations. Hence, a more sensitive method is required to detect the pharmaceutical compounds at low concentrations, and for this; CDs are the best alternative method to detect the pharmaceutical compounds at the femtogram level. Nowadays, nanomaterials can be used for the treatment of wastewater and purification. Materials such as graphene oxide-based NPs (GONPs), mesoporous Mn_x_Co_3-x_O_4_ NPs, etc., have been used for the removal of ibuprofen, ciprofloxacin, and levofloxacin from water and aqueous solution by the mechanism of adsorption mainly due to electrostatic interactions, accelerated electron transfer and by photocatalytic degradation. The percentage removal of ibuprofen, ciprofloxacin, and levofloxacin was found to be 98.2 %, 100 %, and 95 %, respectively [[260], [261], [262]]. CDs with an optimum size of 5 nm–10 nm and modifying the surface functionalization of CDs can be used for various applications apart from bio-sensing. It includes tumor theranostics (cancer-targeted drug delivery, gene delivery, PTT, photodynamic therapy (PDT)), bio-imaging biomarkers (cell membrane, cytoplasm, for self-targeted bioimaging and diagnosis of tumor cells), catalysis, detection of small molecules, photoacoustic imaging [263], light-emitting diode (LED) device [264], FL ink [265], in agriculture [266], etc.

## 5 Concluding remark

This review highlights the recent advancements in CDs, focusing on their synthesis, surface functionalization, photoluminescent properties, and diverse applications across fields such as photocatalysis, energy, and sensing. CDs have emerged as promising alternatives to traditional fluorescent compounds, particularly in in-vivo analysis, due to their safer nature and exceptional FL properties. The unique physical and optical characteristics of CDs, including their efficient electron storage and transport capabilities when exposed to light, present vast untapped potential. Looking forward, the development of more reliable and efficient synthesis methods will likely drive further exploration of innovative applications. The versatility of CDs positions them as impactful materials in biotechnology and environmental remediation, offering safer substitutes for conventional analytical techniques. Given the complexity of contaminants that conventional wastewater treatment plants cannot fully eliminate, there is an increasing need for advanced materials like CDs to address these challenges. With their exceptional electron transfer abilities and light-harvesting efficiency, CDs hold significant promise in photovoltaic and photocatalytic applications. Additionally, their potential extends to areas such as bio-sensing, tumor theranostics, bio-imaging, LED technology, small molecule detection, and agriculture. The continued research and development of CDs are expected to unlock new applications, broadening their utility in various scientific and industrial fields.

## CRediT authorship contribution statement

**Sandesh R. Lodha:** Writing – original draft, Visualization, Conceptualization. **Jesika G. Merchant:** Writing – original draft. **Arya J. Pillai:** Writing – original draft, Visualization. **Anil H. Gore:** Writing – review & editing. **Pravin O. Patil:** Writing – review & editing, Writing – original draft. **Sopan N. Nangare:** Writing – review & editing. **Gajanan G. Kalyankar:** Writing – review & editing. **Shailesh A. Shah:** Supervision, Resources. **Dinesh R. Shah:** Supervision, Resources. **Shashikant P. Patole:** Writing – review & editing, Supervision, Funding acquisition.

## Declaration of competing interest

The authors declare that they have no known competing financial interests or personal relationships that could have appeared to influence the work reported in this paper.
